# Development potential of nanoenabled agriculture projected using machine learning

**DOI:** 10.1073/pnas.2301885120

**Published:** 2023-06-14

**Authors:** Peng Deng, Yiming Gao, Li Mu, Xiangang Hu, Fubo Yu, Yuying Jia, Zhenyu Wang, Baoshan Xing

**Affiliations:** ^a^Key Laboratory of Pollution Processes and Environmental Criteria (Ministry of Education), Tianjin Key Laboratory of Environmental Remediation and Pollution Control, College of Environmental Science and Engineering, Nankai University, Tianjin 300350, China; ^b^Key Laboratory for Environmental Factors Controlling Agro-Product Quality Safety (Ministry of Agriculture and Rural Affairs), Tianjin Key Laboratory of Agro-Environment and Product Safety, Institute of Agro-Environmental Protection, Ministry of Agriculture and Rural Affairs, 300191 Tianjin, China; ^c^Institute of Environmental Processes and Pollution Control, School of Environment and Civil Engineering, Jiangnan University, Wuxi, Jiangsu 214122, China; ^d^Stockbridge School of Agriculture, University of Massachusetts, Amherst, MA 01003

**Keywords:** machine learning, nanoparticle, artificial intelligence, risk, nanoenabled agriculture

## Abstract

The controllability and targeting of nanoparticles (NPs) offer solutions for precise and sustainable agriculture. However, the development potential of nanoenabled agriculture remains unknown. Here, we build an NP-plant database containing 1,174 datasets and predict (*R*^2^ higher than 0.8 for 13 random forest models) the response and uptake/transport of various NPs by plants using a machine learning approach. Multiway feature importance analysis quantitatively shows that plant responses are driven by the total NP exposure dose and duration and plant age at exposure, as well as the NP size and zeta potential. Feature interaction and covariance analysis further improve the interpretability of the model and reveal hidden interaction factors (e.g., NP size and zeta potential). Integration of the model, laboratory, and field data suggests that Fe_2_O_3_ NP application may inhibit bean growth in Europe due to low night temperatures. In contrast, the risks of oxidative stress are low in Africa because of high night temperatures. According to the prediction, Africa is a suitable area for nanoenabled agriculture. The regional differences and temperature changes make nanoenabled agriculture complicated. In the future, the temperature increase may reduce the oxidative stress in African bean and European maize induced by NPs. This study projects the development potential of nanoenabled agriculture using machine learning, although many more field studies are needed to address the differences at the country and continental scales.

## Significance

The development of nanotechnology has enabled precision and sustainable agriculture. The controllability and targeting of nanoparticles (NPs) will accelerate the development of modern agriculture. However, the development potential of nanoenabled agriculture remains unknown. Here, we built models of plant responses and uptake/transport of NPs using machine learning. Feature interaction and covariance analysis provide ideas for the design of environmentally friendly nanoenabled pesticides and fertilizers. The models quantify the synergistic effects among the surface charge, size, temperature, and NP exposure dose on plant growth and NP uptake. According to the prediction, Africa is a suitable area for nanoenabled agriculture, where a moderate temperature increase (approximately 6.0°) in the future may reduce the oxidative stress of bean induced by NPs.

Nanoparticles (NPs) or nanomaterials are incorporated into a wide range of agricultural fields because of their extraordinary nanoscale properties (e.g., high reactivity, mobility, and biocompatibility) (1, 2). For example, nanotechnology is applied to enhance soil fertility by supplying nutrients (nanofertilizers), controlling weeds and pests (nanoherbicides and nanopesticides), and remediating and improving soil properties and plant characteristics (3–6). Nanotechnology for agricultural applications is undergoing increasing development and application (7), but development is still at an early stage (1). However, there is substantial uncertainty regarding nanoenabled agriculture, such as the adverse effects of NPs, the cost and the development potential at the global scale (2, 8). This uncertainty is affected by the types of NPs, plant species, and environmental and climate conditions (3). With the increasing global population and food security issues, it is urgent to develop smart methods to assess the above uncertainty, promote nanoenabled agricultural development and reduce risks to plant growth (9, 10).

Previous studies focused on experiments that evaluated the effect of NPs on plant growth, toxicity, translocation, and accumulation, and these studies were expensive and time-consuming and had low reproducibility (2, 11). According to the physicochemical properties of NPs and the plant growth environment, the rapid and accurate prediction of their growth, responses, translocation, and accumulation in plants is urgently necessary for the development of nanoenabled agriculture (12, 13). Traditional methods [e.g., quantitative structure-activity relationship (QSARs) and molecular dynamics simulations] are conducted at the atomic level, and it is difficult to accurately predict the systemic toxicity response and translocation and accumulation of NPs in fields and at a large scale (14, 15). As data-driven approaches, machine learning models present a robust ability to reveal complex relationships and project future trends (16, 17). Currently, quantitative predictions of the fate of NPs in plants under environment-related and large-scale conditions remain inaccessible (18). The lack of high-quality datasets affects the effective training, testing, and verification of models for the interactions between NPs and plants. To address these problems, we built a database containing 1,174 datasets on NP-plant responses and uptake/transport from different countries or regions. To further screen the key factors driving the interactions between NPs and plants, we improved machine learning models to overcome the challenges of the high dimensionality and heterogeneity of data through the visualization of factor covariance.

**Fig. 1.**
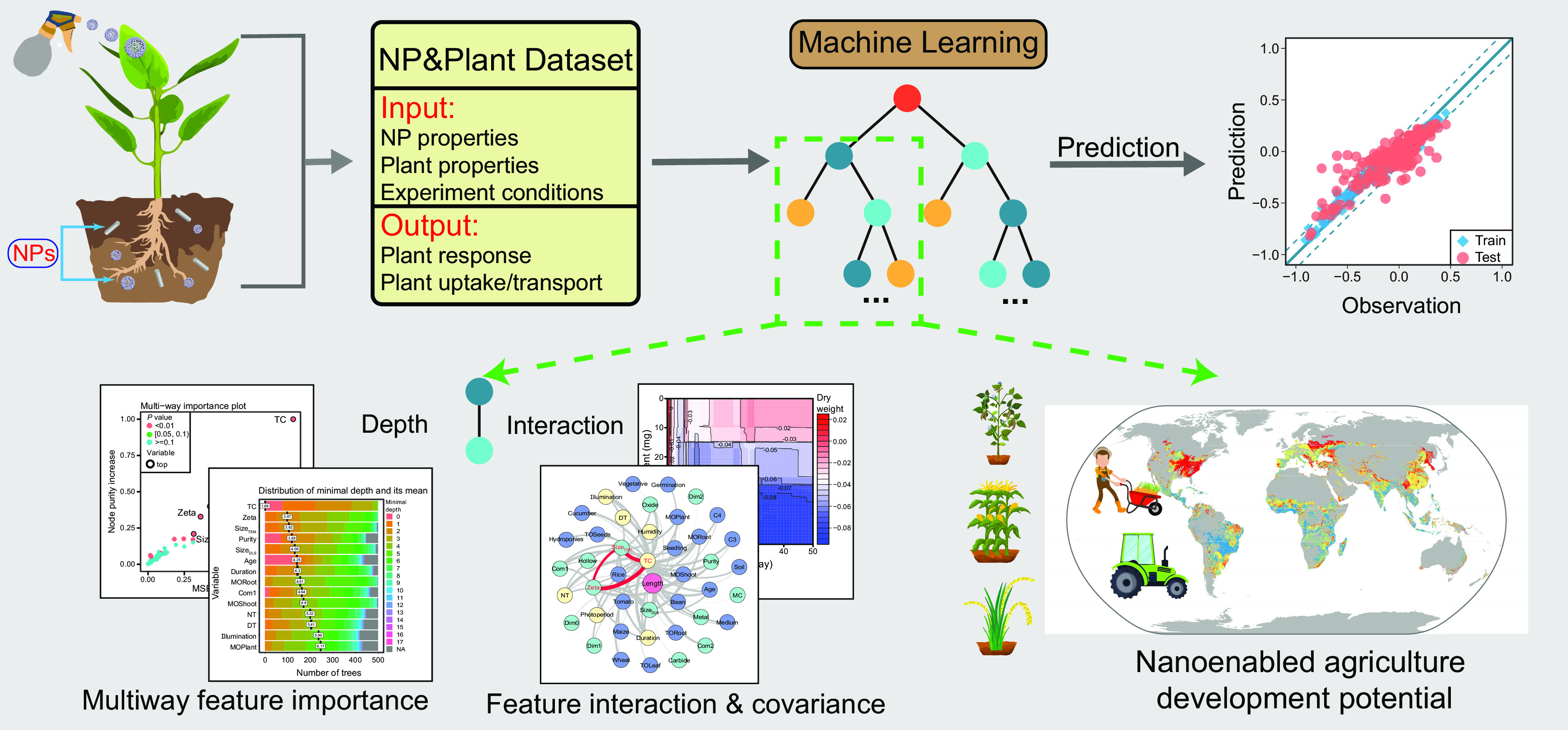
Workflow for studying the development potential of nanoenabled agriculture projected using machine learning with laboratory and field data. Reliable datasets are established through the collection of database data from the literature. The optimized machine learning models include RF feature selection, multiway feature importance and feature interaction and covariance analysis. The development potential of nanoenabled agriculture is projected using the established machine learning models based on both laboratory and field data.

Although the potential of nanotechnology in agriculture has been discussed for more than a decade, it has not been very rapidly implemented in practical use (19). Assessments of plant responses and the uptake of specific NPs at multiple geographic sites in actual soil and climatic environments are critical for the development of nanopesticides and fertilizers at local and global scales. Here, we compiled high-spatial-resolution laboratory and field data on soils (acidic, neutral, and alkaline), climate (daytime and night temperatures, illumination intensity, and relative humidity) and local crop planting dates and predicted crop growth, oxidative stress, photosynthesis, and the uptake of NPs, illustrating the development potential of nanoenabled agriculture on different continents and in different countries (Fig. 1 and *SI Appendix*, Fig. S1). Quantitative analysis of variable covariance and prediction at the local and global scales using machine learning and the integration of laboratory and field data will boost the development of precision and sustainable nanoenabled agriculture.

## Results and Discussion

### Heterogeneity Challenge Analysis of NP–Plant Interactions.

**Fig. 2.**
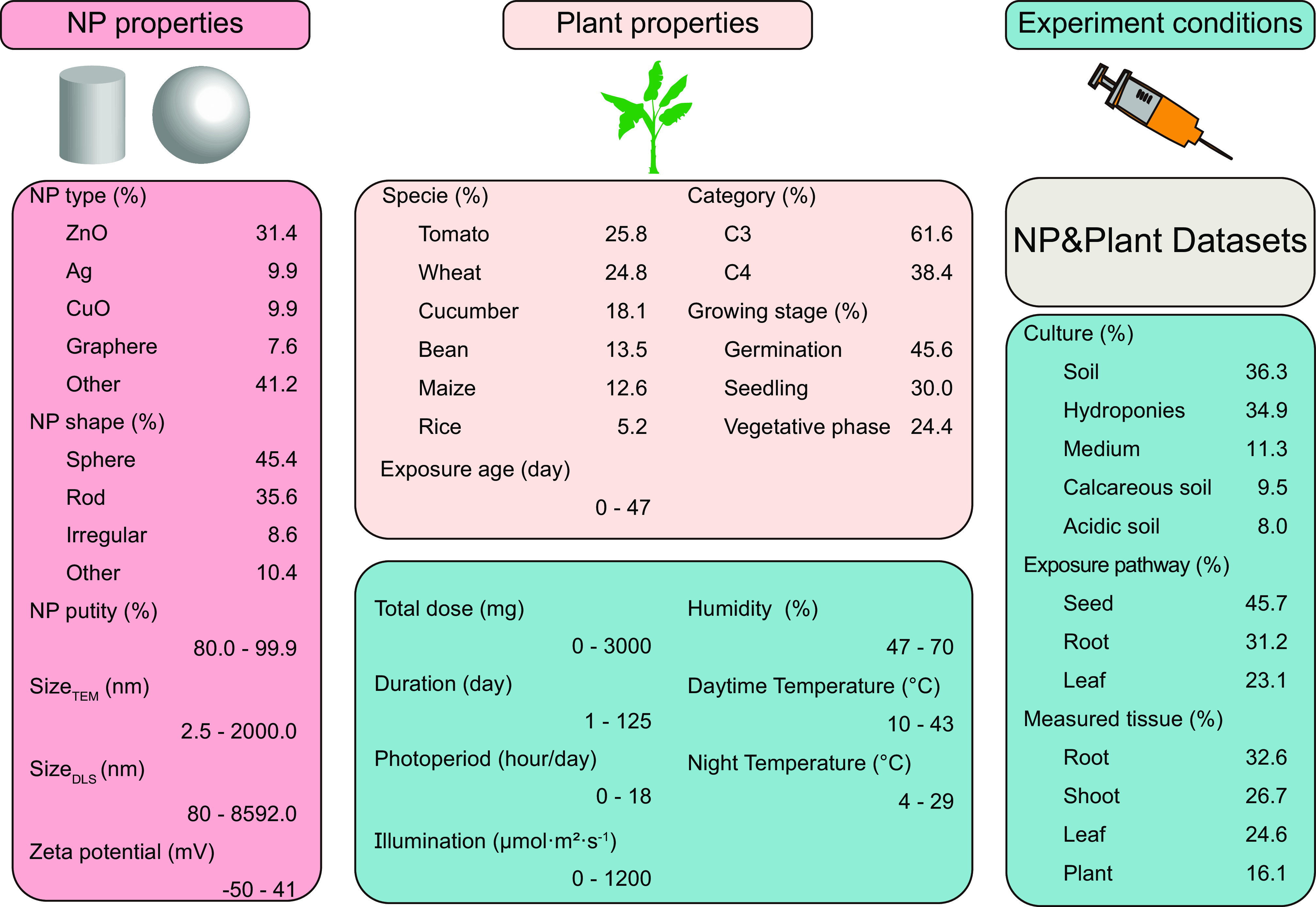
Overview of the datasets (*n *= 1,174) of the qualitative and quantitative factors determining the interactions between NPs and plants. The difference between the C3 and C4 plants is the photosynthesis system. C3, photosynthetic performance in mesophyll cells; C4, photosynthetic performance in mesophyll cells and bundle sheath cells. The exposure pathway refers to seed exposure, root exposure and leaf exposure. Measured tissue refers to the plant tissue that was measured for the biological responses.

Current studies on the effects of NPs on plants are incomplete and have been carried out under specific conditions (e.g., one type of NP with one exposure pathway to expose one specific plant growth condition). A systematic database of various NPs acting on different plants is lacking, preventing a global examination of NP–plant interactions. There is an urgent need to use machine learning to extract and mine hidden response relationships of plants to NPs to provide technical support for nanoenabled agriculture. To reduce the data bias caused by different study conditions and ensure the quality of the data and the reliability of the results (20, 21), strict standards were adopted in literature extraction and data mining (described in the *Methods*). A total of 1,174 datasets related to the various NPs in plants from different countries or regions were mined and analyzed (*SI Appendix*, Fig. S2). The multidimensional factor distribution of the datasets is shown in Fig. 2 and *SI Appendix*, Table S1. The 17 unique NPs included metallic (e.g., Ag, CuO, and ZnO), carbonaceous (graphene and multiwalled carbon nanotubes), nonmetallic (SiO_2_), and macromolecular polystyrene NPs. The quantitative factors represented highly heterogeneous (e.g., 2.5 to 2,000.0 nm for NP size measured by transmission electron microscopy [size_TEM_] and 0 to 3,000 mg for NP exposure doses) and complex (e.g., relationships between 20 input factors and 13 output labels) conditions for the prediction of the interactions between NPs and plants.

Data complexity (e.g., high heterogeneity) was the main challenge in accurately predicting these interactions using traditional statistical methods (e.g., multiple linear regressions) and machine learning models (22). We used the similarity network based on proximity matrixes from random forest (RF) models to visualize the heterogeneous distribution of NP types and shapes, as shown in *SI Appendix*, Fig. S3. The high homogeneity of the tight-knit clusters revealed similar factor-response relationships hidden in the RF models. In various NP types (*SI Appendix*, Fig. S3 *A*–*D*), tight connections are present in the clusters of ZnO, Ag, CuO, and carbon-based NPs. More connected nodes indicate NPs that share more similar factor-response dependence or NP–plant acting patterns, and vice versa. The dissolution of metal-oxide NPs in plants (depending on the specific metal properties) plays an important role in their biological impact, while carbon-based NPs are prone to oxidative degradation by hydroxyl radicals, leading to different clustering compared with metal NPs (8, 23). In addition, clear tight connections are present in the clusters of NP shapes (*SI Appendix*, Fig. S3 *E–H*). The above similarity network identified data homogeneity and heterogeneity and supported the subsequent processing of data heterogeneity for the same NPs rather than different NPs with similar effects. Given the sharp increase in the dimensionality and data heterogeneity resulting from the type and shape of the NPs in the RF model, we used two coding methods (NP type and shape) to represent these two features of NPs (shown in the *Methods*). This approach reduces the complexity of the data and enhances the generalization ability of the model.

### Model Optimizations for NP–Plant Interactions.

As shown in *SI Appendix*, Fig. S4, most of the multiple linear regressions had low correlation coefficients [coefficient of determination (*R*^2^), 0.56 ± 0.15] and high rms errors (5.37 ± 5.76). In contrast, robust machine learning methods such as RF, extreme gradient boosting (XGB), support vector machines (SVMs), and artificial neural networks (ANNs) can handle complex and heterogeneous data (22). To eliminate dimensional effects and balance the weights of features, *z score* normalization and encoding of the character variables were applied prior to model training (details are provided in *Methods*). The distribution ranges of the label data were very wide [e.g., ascorbate peroxidase ranged from 5.30 to 941.58% (control group set to 100%)], and a considerable number of outliers were present. Therefore, the label values were normalized to improve the accuracy of the models. To avoid overfitting and to credibly evaluate the prediction accuracy, the model performance was estimated by 10-fold cross-validation (21). The *R*^2^ of the regression (*SI Appendix*, Fig. S5) shows that for the test set, the performance of the RF model (average of all models, 0.82 ± 0.14) was better than that of XGB (average of all models, 0.80 ± 0.18), ANN (average of all models, 0.71 ± 0.17) and SVM (average of all models, 0.69 ± 0.18). *SI Appendix*, Fig. S6 and Table S2 list the RF regression results. The *R*^2^ values of the test set of most RF models were greater than 0.75, where the *R*^2^ values for length, root-shoot ratio (RS ratio), and uptake were >0.85, and the maximum value reached 0.88. Sequential backward selection (SBS, described in *Methods*) was used to remove redundant features and simplify the models. *SI Appendix*, Fig. S5*E* shows that no evident improvement in the model performance was obtained through feature selection by the SBS algorithm, and the SBS algorithm ignored some NP properties. Therefore, we chose the full features to build RF models for the subsequent analysis.

 *SI Appendix*, Fig. S7 shows that most of the features had low linear correlations, indicating that the features obtained via the literature and generated through coding may not have caused overfitting due to multicollinearity. The biological interaction of NPs in plants is complex and unclear, and a single factor cannot provide sufficient information for the response and uptake/transport (3). Moreover, we performed permutation tests, and the intercepts of the cross-validation coefficients (*Q*^2^) on the *Y *axis were all less than 0.05, indicating that the models did not overfit (*SI Appendix*, Fig. S8) (17). Furthermore, we performed feature value shuffling. The predictive performance was abrogated after feature value shuffling (*SI Appendix*, Fig. S9), ensuring that the features contributed valid information to the models.

### Screening Drivers and Covariance Analysis of NP-Enabled Agriculture.

**Fig. 3.**
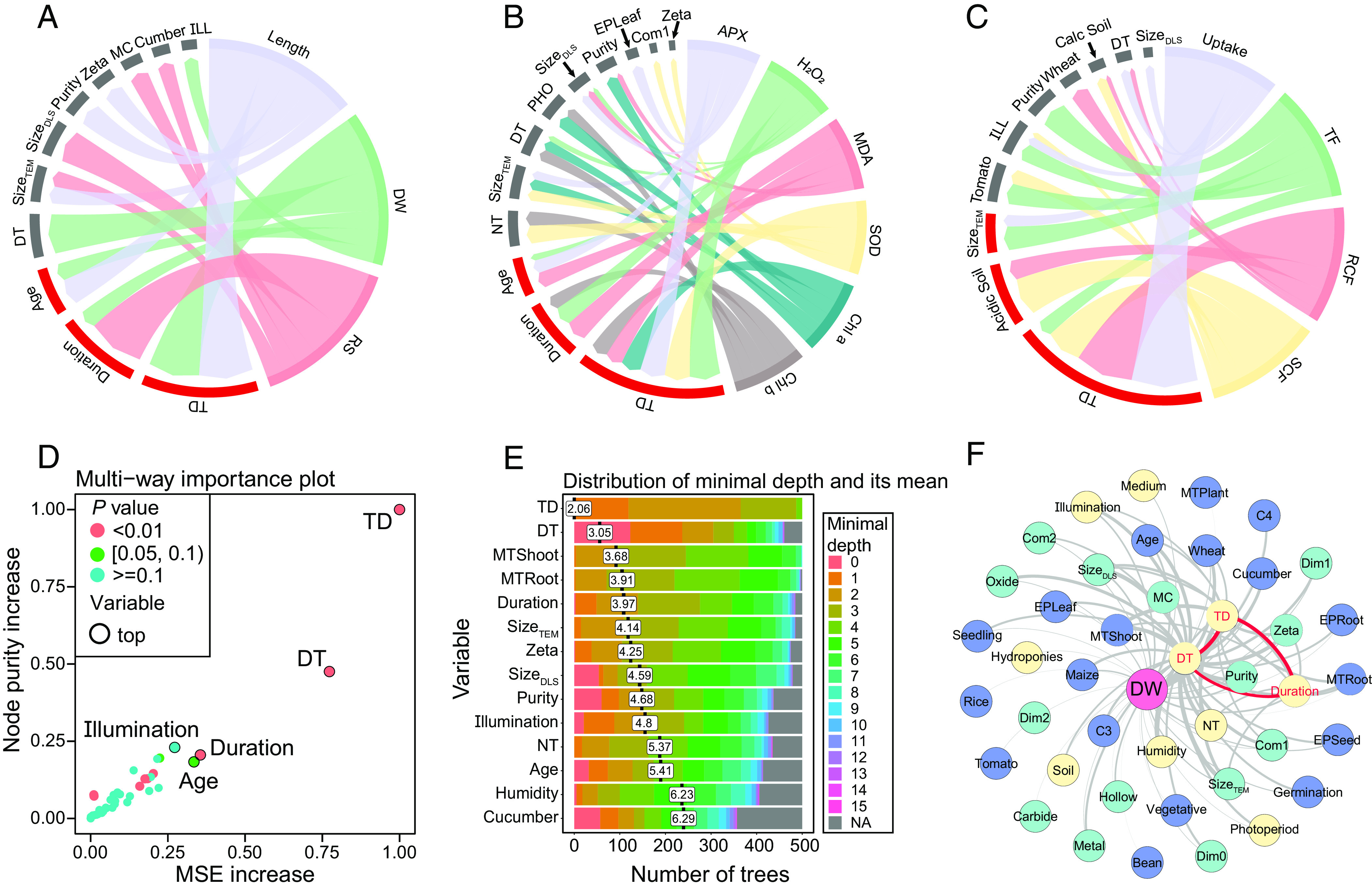
Feature importance and interaction analysis. (*A*–*C*) Top five important features of each indicator measured by the MSE increase. ILL, illumination intensity; PHO, photoperiod; Calc Soil, calcareous soil. The width of the ribbons connecting the feature and indictor represents the importance. The red segment represents relatively important features of each indicator. *A*, Growth response. *B*, Oxidative stress and photosynthesis response. *C*, NP uptake/transport. The “uptake” represents the amount of NPs taken up by plants after NP exposure, in units of mg/g. (*D*) Multiway feature importance analysis of the plant dry weight model combining the MSE increase, node purity increase, and *P* values of the features. “Duration” refers to the time elapsed from the exposure of the plant to NPs to the measurement of the dry weight of the plant. (*E*) Distribution of the features’ mean minimal depth, where the total NP exposure dose, daytime temperature, and measured tissue (shoot) are closer to the root of the trees than the other features. The width of the color band represents the number of decision trees for each minimum depth value of the feature. The numbers in the white boxes represent the mean minimum depth value for each feature. (*F*) Feature interaction network for plant dry weight based on the number of occurrences. Nodes of different colors represent different types of features and indicators. Green, NP properties; blue, plant properties; yellow, experimental conditions; and pink, response index. The line between the two features represents the top 50 interaction strengths based on the number of occurrences. The thicknesses of the lines represent the strengths of the interactions. The three strongest NP properties and experimental condition interactions in the networks are highlighted in red. TD, total NP exposure dose; DT, daytime temperature; NT, night temperature; MC, macromolecular compound; EP, exposure pathway; MT, measured plant tissue.

 *SI Appendix*, Fig. S10 shows the importance of features based on the mean square error (MSE) increase and node purity increase (22). Fig. 3 *A–C* show the top five important features of each plant label measured by the MSE increase. The total NP exposure dose was the priority driver of the plant response and NP uptake/transport. Multiway feature importance analysis was used to screen other important drivers. The total NP exposure dose, daytime temperature, and duration were identified as the most important factors that affected the plant dry weight (Fig. 3 *D* and *E*). Multiway feature importance analysis avoided the bias of a single indicator (Fig. 3 *D* and *E*). Notably, the plant length was mainly driven by the NP properties (e.g., zeta potential, size_TEM_, and purity), as shown in *SI Appendix*, Fig. S11. Based on the oxidative stress response, plant age should be considered a priority factor for NP exposure (*SI Appendix*, Figs. S12–S15). Partial dependence analysis (*SI Appendix*, Fig. S16*B*) showed that oxidative stress decreased with increasing duration, and NPs tended to induce short-term acute oxidative stress in plants (e.g., tomato and wheat). The oxidative stress indicators increased rapidly in the initial exposure stage. Within approximately 20 d after exposure, hydrogen peroxide activity decreased from 133.3 to 114.5% of that in the control group (corresponding to the normalized values of 0.250 and 0.127, respectively, in *SI Appendix*, Fig. S16*B*). Plants’ exposure at approximately 25 d (the transition stage from the seedling to vegetative phase) caused malondialdehyde to increase rapidly. Hence, the increased oxidative stress in the initial exposure stage deserves attention for the applications of NPs. The decrease in night temperature (< 15 °C) inhibited photosynthesis, as shown in *SI Appendix*, Fig. S16*C*. According to the RF models, exposure to 60 mg of the 17 tested NPs increased the average chlorophyll *a* content by 7.6% in the test plants and improved the low temperature tolerance (*SI Appendix*, Fig. S16*C*). The NP exposure dose is well known as a priority driver of uptake, and *SI Appendix*, Figs. S17 and S18 show that the uptake and transport factor (TF) of NPs in plants was size specific. The NP size affected the uptake of NPs in the plant leaf epidermis via stomata, as well as the translocation across the plant plasma membrane and organelle lipid bilayers in vivo (24, 25). Importantly, the uptake and transport of NPs depend on the plant species (26). Plant species and culture type affected the root concentration factor (RCF) and shoot concentration factor (SCF) of NPs in the plants (*SI Appendix*, Figs. S19 and S20). We conducted significance tests on the plant uptake and transport data in different plant species in *SI Appendix*, Fig. S21, which showed significant differences in uptake, TF and SCF among the five tested species. The ZnO NP dose in wheat roots was higher than that in other plants, probably because ZnO NP induced the formation of the lateral roots of wheat to promote NP uptake (27). With respect to ZnO NP exposure, maize had a lower TF value than the other plants, probably due to its high capacity to store Zn in roots (27).

**Fig. 4.**
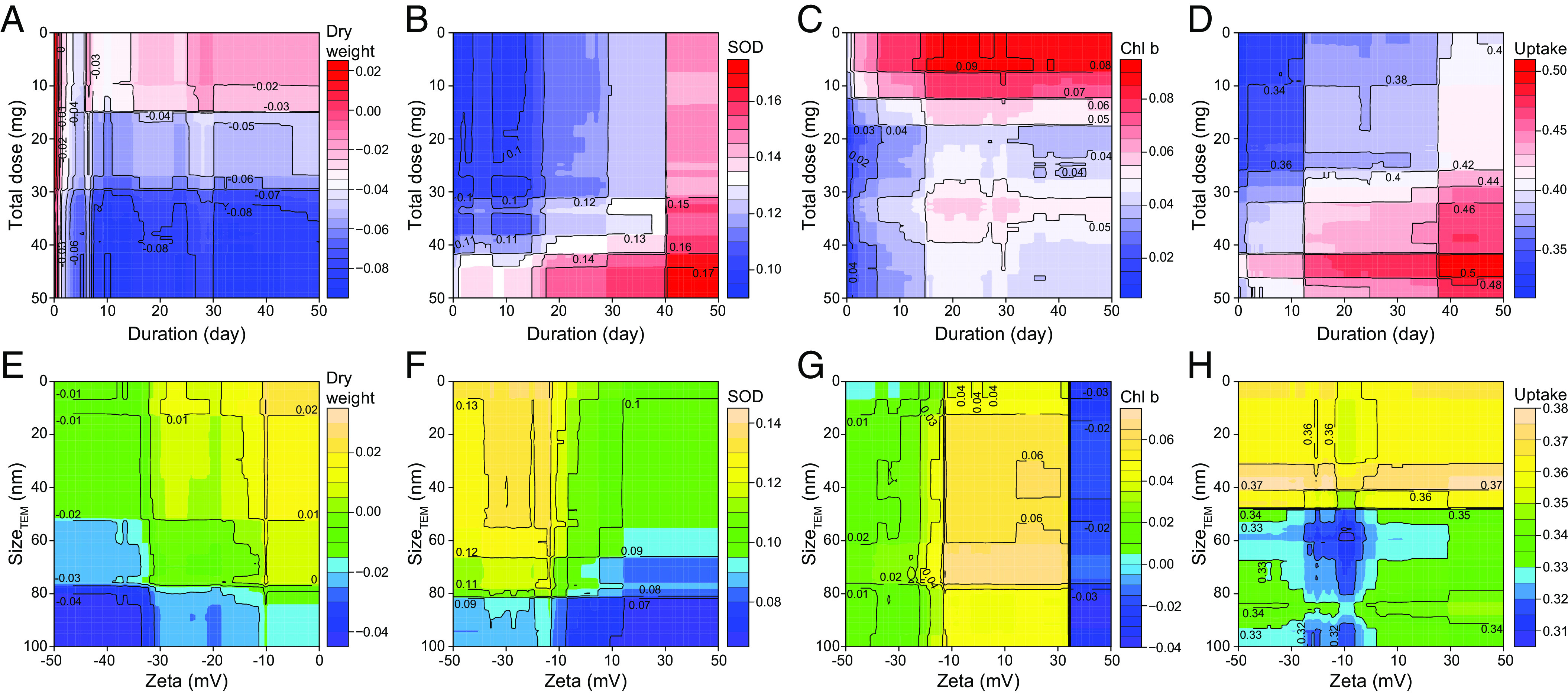
Double-variable partial dependence for the total exposure dose-duration and size_TEM_-zeta potential. (*A and *E**) Dry weight; (*B and *F**) superoxide dismutase; (*C and *G**) chlorophyll *b* and (*D and *H**) NP uptake.

Understanding how NPs interact with plants and affect their responses and uptake/transport is critical for precision and sustainable nanoenabled agriculture (7). However, related information remains largely unknown due to the complicated interaction between NPs and plants (28). Most machine learning models, such as black-box models, are not well suited for further research on feature interactions (17). We calculated the feature–feature interaction strength based on the structure of each tree in the RF model and established the feature interaction networks (described in the *Methods*). The dry weight interaction network is shown in Fig. 3*F*, and other networks are presented in *SI Appendix*, Figs. S11–S15, S17–S20, and S22–S24. The plant response network analysis indicated that size (size_TEM_ and size_DLS_) had strong interactions with the zeta potential of the NPs, while the NP exposure dose had strong interactions with duration and temperature (daytime and night temperature). According to the plant uptake/transport network analysis, the NP exposure dose interacted strongly with the culture substrate (e.g., acidic soil). The strong NP exposure dose–duration and size_TEM_–zeta potential interactions were displayed by double-variable partial dependence analysis in Fig. 4. For example, under a low NP exposure dose (<15 mg), the tendency of NPs to inhibit plant growth gradually weakened with duration, while a high NP exposure dose (>30 mg) caused irreversible damage to plants as the duration increased (29) (Fig. 4*A*). Fig. 4 *C* and *D* suggests the feasibility of achieving optimized effects by controlling the NP exposure dose and duration, such as enhancing photosynthesis and uptake. The zeta potential and size of NPs also played important roles in the formation of protein crowns, which affect uptake by plants (30, 31). For example, Fig. 4*E* shows that low negative charges (−20 to 0 mV) with a small size_TEM_ (0 to 20 nm) of NPs promoted plant growth. NPs with a negative charge (<0 mV) and small size_TEM_ (0 to 50 nm) induced an average 12.4 to 15.0% increase in superoxide dismutase activity, as shown in Fig. 4*F*. Superoxide dismutase activity resists superoxide radicals (32), and a suitable zeta potential (<−10 mV) increases superoxide dismutase activity and may resist damage from superoxide radicals. For NPs with a size_TEM_ less than 50 nm in Fig. 4*H*, the zeta potential had a limited effect on NP uptake by plants (e.g., cucumber). However, for NPs (e.g., Fe and ZnO) ranging from 50 to 100 nm, high surface charges (zeta potential >20 mV or <−20 mV) promoted NP uptake by plants. The above results are consistent with the previous proposal that a high surface charge is conducive to the realization of trans lipid membrane translocation in maize (33, 34). For the design and application of nanopesticides or other NPs, sizes and zeta potential should be considered simultaneously to achieve controllable uptake.

### Development Potential of Nanoenabled Agriculture Projected by Models.

**Fig. 5.**
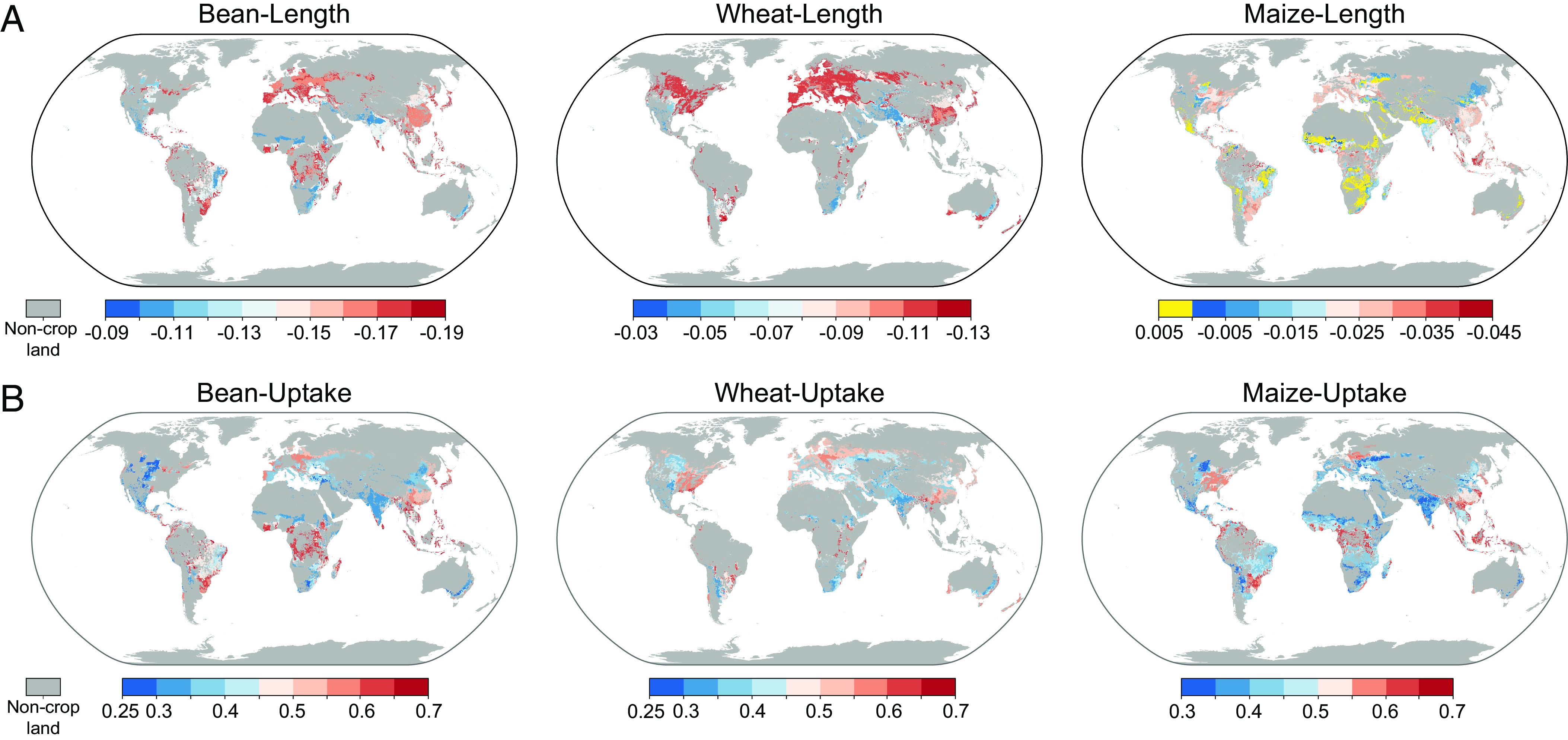
Spatial patterns of plant root length regulated by NP and NP uptake projected by models with laboratory and field data. (*A*) The effect of 50 mg Fe_2_O_3_ NP on the crop root length during the planting period. (*B*) ZnO NP (50 mg) uptake by crop roots during the planting period. The spatial patterns are predicted with RF models. Detailed feature settings are shown in *SI Appendix*, Table S8. Values were transformed and are unitless and are shown in the *Methods*. The map has a spatial resolution of 5 arcmin, which is approximately 10 km × 10 km at the equator. The bean, wheat, and maize distributions are from the global field crop planting dataset (44).

Previous studies have focused on specific case studies, whereas local- and global-scale assessments of plant responses and NP uptake have been lacking, thus hindering the development of nanoenabled agriculture. This study attempted to fill the gap between laboratory research and field applications. Based on the above established models, we selected Fe_2_O_3_ and ZnO NPs, which are the most commonly used NPs in agriculture (28, 35), and projected their development potentials (effects on growth, oxidative stress, and uptake) in three widely distributed crops (bean, wheat, and maize) (Fig. 5). The data from laboratory experiments (e.g., daytime and night temperatures, soil type, illumination intensity, and relative humidity) corresponded one-to-one with the field features in the models. We found that daytime temperature was the main environmental factor of the low growth inhibition of bean root length induced by Fe_2_O_3_ NP (blue regions), and relative humidity was the main environmental factor of high growth inhibition (red regions) in Fig. 5*A* and *SI Appendix*, Fig. S25*A*. The detailed factors for differences in growth inhibition in Europe are provided in Fig. 5*A*. Beans presented more severe growth inhibition by Fe_2_O_3_ NPs (84.3%, corresponding to the normalized value of −0.157 in *SI Appendix*, Fig. S26*A*) in Europe compared with other continents, possibly due to the synergism of low night temperatures (*SI Appendix*, Table S3) and NPs, with 49.5% of bean areas mainly driven by night temperature (*SI Appendix*, Fig. S25*A*). Compared with other continents, in Europe and North America, wheat presented high risks of inhibition (*P* < 0.01) in *SI Appendix*, Fig. S26*A*, with 39.1% and 47.7% of the respective regions mainly driven by night temperature in *SI Appendix*, Fig. S25*B*. Low-temperature stress disrupts plant growth processes (36). According to the above results, Fe_2_O_3_ NP may aggravate the effect of low-temperature stress on wheat root growth (*SI Appendix*, Table S4). Compared to other continents, Africa had lower root growth inhibition (−0.013), as shown in Fig. 5*A*. Fe_2_O_3_ NP has been proven to improve the photosynthesis of maize seedlings with increased chlorophyll and carotenoid contents under high-temperature stress (37, 38). Fe_2_O_3_ NP has potential application in maize cultivation in Africa with high daytime temperatures. Daytime temperature and night temperature were the main environmental factors of the uptake of ZnO NP by crop roots during the seedling stage (*SI Appendix*, Fig. S27). The root uptake capacity of beans in Oceania (0.361) and North America (0.381) during the seedling stage was weak compared with that of other continents, which was related to the high daytime temperature in Fig. 5*B*. High-temperature stress may reduce the root function of seedlings by altering root system architecture (e.g., root depth and root width) and gene expression (e.g., inhibition of Pi starvation-related gene expression decreased the level of Pi in roots) to reduce the ability of crop roots to take up NPs (39–41). High illumination intensity (523.3 μmol m^−2 ^s^−1^) in maize regions (6.1%) in Latin America promoted the root uptake of ZnO NP (*SI Appendix*, Figs. S26*B* and S27*C*). High illumination intensity enhanced photosynthesis to promote maize root growth (e.g., root surface area and root hairs) and then indirectly improved the uptake of ZnO NP (42, 43).

**Fig. 6.**
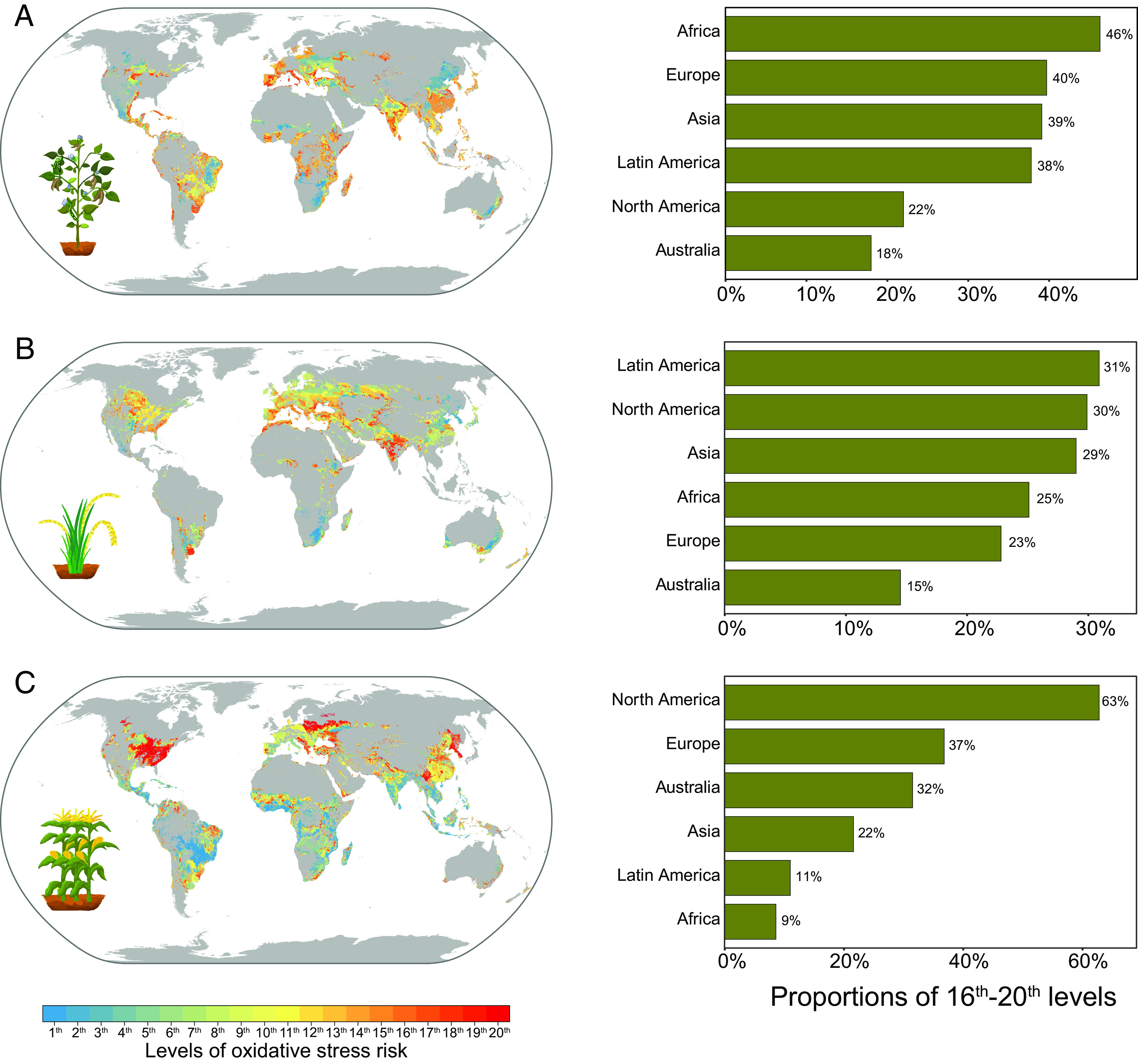
Spatial patterns of crop oxidative stress risk levels associated with NPs projected by models with laboratory and field data. The effect of ZnO NPs (50 mg) on the oxidative stress of the crop leaves during the planting period. (*A*) Bean. (*B*) Wheat. (*C*) Maize. The histograms show the percentage of the areas at the 16th to 20th level of risk on the global map. Oxidative stress responses were predicted with RF models. Detailed feature settings are shown in *SI Appendix*, Table S8. The map has a spatial resolution of 5 arcmin, which is approximately 10 km × 10 km at the equator.

This study projected the oxidative stress risk levels of NPs for crops in the fields by integrating a reliable machine learning model and laboratory and real field data (Fig. 6). The low oxidative stress during the bean seedling stage in Oceania (18% of oxidative stress at the 16th to 20th level) was projected compared to other continents (Fig. 6*A*). The illumination intensity (584.9 μmol m^−2 ^s^−1^) was the main environmental factor of the high oxidative stress in bean leaves induced by ZnO NP in Oceania (red regions), as shown in Fig. 6*A* and *SI Appendix*, Fig. S28*A*. ZnO NP aggravated the effect of high illumination intensity stress on bean leaf oxidative stress. Compared to other continents, North America had a high oxidative stress risk (63% of oxidative stress at the 16th to 20th level) induced by ZnO NP, as shown in Fig. 6*C*, with 62.1% of the regions mainly driven by night temperature, as shown in *SI Appendix*, Fig. S28*C*. Maize is sensitive to low-temperature stress during the seedling stage (45–47). The low-temperature stress in North America is shown in *SI Appendix*, Table S5. The above results agree with the previous proposal that environmental conditions in specific regions play a critical role in the application of nanopesticides (1). In Africa, compared to bean and wheat, the regions where maize was grown under oxidative stress at the 16th to 20th level were small (9%) (Fig. 6). Nanopesticides based on ZnO NP may have potential applications for maize in Africa with high night temperatures. Based on regional differences in climate conditions (44), machine learning models predicted the global response and uptake of NPs during the crop seedling stage and identified environmental factors, although more field research is needed to validate the above results (48). The differences between continental and national scales provide optimization insights for nanoenabled agricultural applications, although many factors (such as the safety profile and cost) should be considered together (49, 50).

Climate change, particularly temperature increases, has resulted in great challenges to modern agriculture (51, 52). The important feature analysis and Shapley value suggested that temperature was a critical factor of the interactions between NPs and plants. Plant responses and the uptake of NPs were predicted for four future scenarios in 2080 to 2100 (*SI Appendix*, Figs. S29–S31) to explore the effect of NPs on plants under temperature increases. During the seedling stages of bean and wheat, the temperature increase led to an increase (SSP1, approximately 1.9 °C for bean and 2.1 °C for wheat compared with now) in root growth inhibition followed by a decrease, while the inhibitory effect of Fe_2_O_3_ NP on maize roots was alleviated after the temperature increase (*SI Appendix*, Fig. S29). An appropriate temperature increase may offset the adverse effects of Fe_2_O_3_ NPs on plant root growth (53). The temperature increase in the SSP5 scenario (approximately 6.0 °C for beans and 5.9 °C for maize compared with now) reduced the oxidative stress in African beans and European maize induced by NPs (*SI Appendix*, Fig. S31). In contrast, a previous study proposed that increased temperature significantly enhanced the toxicity of nanooxides to wheat by interfering with metabolism (e.g., disturbance of energy metabolism) (54), and more reliable research on the synergism of NPs and temperature is needed for the practical application of nanoenabled agriculture (55). The above findings reveal the challenges posed by global regional differences and temperature changes to nanoenabled agriculture. The selection of typical fields for NP application is urgent to verify the above results for the development of nanoenabled agriculture.

## Conclusion

Here, we built machine learning models to successfully identify and predict the interactions between NPs and plants. Machine learning models were used to analyze plant responses and NP uptake driven by NP properties and climatic and environmental factors. The feature interaction network revealed hidden relationships to provide global insights into the plant responses induced by the NPs and their uptake/transport. The feature covariance analysis explained the critical roles of NP size_TEM_ and zeta potential in the plant responses and NP uptake; for example, NPs with a low negative charge (−20 to 0 mV) and a small size_TEM_ (0 to 20 nm) tended to promote plant growth. The established models predicted the responses and NP uptake in typical crops around the world and revealed the synergism between low night temperatures and NP exposure that may pose severe bean root length inhibition by ZnO NPs in Europe. The oxidative stress risks for maize were lower than those for bean and wheat in Africa due to the synergism between high night temperatures and ZnO NP exposure. Based on the prediction in the four future scenarios in 2080 to 2100, the temperature increase may reduce the oxidative stress in African bean and European maize by NPs. Currently, there are no comprehensive studies that evaluate plant responses to NPs under field conditions (56). This is a crucial knowledge gap between laboratory research and actual applications, leading to uncertainty in models. The uncertainty analysis of the projected global plant length, uptake, and oxidative stress prediction is provided in *SI Appendix*, Fig. S32. The models may not be perfect for complicated systems at a large scale or local regions due to the imitated data but will be improved with the increase in field studies (57). There are many debates related to the toxicity of NPs at environmentally relevant concentrations, especially in soil (58), which impedes the development of nanoenabled agriculture. Scientific judgements and applications of NPs are urgent for the public (59). Machine learning that integrates laboratory and field studies will promote the development of nanomaterials in modern agriculture.

## Methods

### Data Extraction.

The publications in Institute for Scientific Information Web of Science, ScienceDirect, and Springer-Link were searched on 31 December 2021 using the following key words: topical subject (TS) = (nano*), TS = (plant or vegetable or rice or wheat or *Cucumis sativus* or cucumber or tomato or bean or corn or maize), and TS = (phytotoxic* or agriculture). The search initially identified 6,055 studies. Given the heterogeneity of the data, 57 studies were selected based on the following criteria: i) The full text was available; ii) the exposure experiment involved both experimental and control groups; iii) basic nanomaterial characterization data and experimental conditions were provided; and iv) indicators reflecting plant growth, plant physiological, and biochemical status and NP uptake were reported. The names and DOI numbers of the 57 studies are provided in Dataset S1. The global geographical distribution of the samples is shown in *SI Appendix*, Fig. S2. Finally, a total of 1,174 datasets were extracted and screened to establish the database. The size of the datasets could support modeling and subsequent analysis, and similarly sized datasets have been successful in studies of the protein corona (1,219 samples) and resistance genes (1,088 samples) (21, 60). Datasets were all from laboratory data. The studies were from 2012 to 2021. To accurately and comprehensively establish the relationship between NP exposure characteristics and plant responses, the dataset consisted of 20 features covering NP properties, environmental factors, and experimental conditions. Three growth indicators (organ length, dry weight, and RS ratio), six antioxidant responses and photosynthesis indicators (ascorbate peroxidase, hydrogen peroxide, malondialdehyde, superoxide dismutase, chlorophyll *a,* and chlorophyll *b*), and four uptake/transport indicators (uptake, TF, RCF, and SCF) were selected as the output labels representing the development potential of nanoenabled agriculture (*SI Appendix*, Table S6). The RS ratio represents the ratio of the fresh weight of the roots to the fresh weight of the shoots. TF describes the ability of NPs to be transferred from roots to shoots within plants. RCF and SCF represent the capacity of plant roots and shoots, respectively, to take up NPs from the growth media (18, 61). The three indicators were defined as shown below:

[1] $$TF=\frac{Concentration\;of\;NPs\;in\;plant\;shoots\;\left(\mathrm{mg}/\mathrm{kg}\;\mathrm{dry}\;\mathrm{weight}\right)}{Concentration\;of\;NPs\;in\;plant\;roots\;\left(\mathrm{mg}/\mathrm{kg}\;\mathrm{dry}\;\mathrm{weight}\right)},$$

[2] $$RCF=\frac{Concentration\;of\;NPs\;in\;plant\;roots\;\left(\mathrm{mg}/\mathrm{kg}\;\mathrm{dry}\;\mathrm{weight}\right)}{Concentration\;of\;NPs\;in\;the\;growth\;media\;\left(\mathrm{mg}/L\right)\;or\;\left(\mathrm{mg}/\mathrm{kg}\right)},$$

[3] $$SCF=\frac{Concentration\;of\;NPs\;in\;plant\;shoots\;\left(\mathrm{mg}/\mathrm{kg}\;\mathrm{dry}\;\mathrm{weight}\right)}{Concentration\;of\;NPs\;in\;the\;growth\;media\;\left(\mathrm{mg}/L\right)\;or\;\left(\mathrm{mg}/\mathrm{kg}\right)}.$$

The data on the plant responses to NPs are complex and are spread across the text, tables, and graphs of publications. Machine extraction of the required data is difficult. Data extracted from text and tables were copied manually. For the data in graphs, three readings were taken for each point using the “digitizer” tool provided by Origin Lab (2021, USA), and the average values were then calculated. The full terms and abbreviations included in the dataset are listed in *SI Appendix*, Table S7.

### Data Preprocessing.

The eight characteristic variables (NP type, shape, plant species, plant category, plant growing state, plant culture, exposure pathway, and measured plant tissues) in the datasets were coded. The exposure pathway refers to seed exposure, root exposure, and leaf exposure. Plant tissues, including roots, shoots, leaves, and plants, were measured. One-hot is a general encoding method that converts unordered discrete variables into binary vectors to overcome the problem of machine learning algorithms failing to recognize these variables (62). The “one-hot” coding adopted the six discrete features (plant species, plant category, plant growing state, plant culture, exposure pathway, and measured plant tissues). There was no obvious correlation between the features obtained by the one-hot coding (*SI Appendix*, Fig. S7). During RF model building, discrete features were one-hot encoded. In the raw dataset, there were 17 and 6 classifications for NP type and shape, respectively. To avoid the sharp increase in the dimensionality and to increase the generalization ability of features, we used two coding methods (type coding method and shape coding method) based on the physicochemical properties to describe the two features (NP types and shapes), as listed below. Other properties (e.g., zeta potential, purity and size_TEM_) were uniformly described and quantized and did not need to be coded. To reduce the biases caused by the imbalance of NP types, the type coding method converted discrete NP types into continuous features. The two coding methods were as follows:

NP types: carbide (0/1), metal (0/1), oxide (0/1), macromolecular compound (M.C., 0/1), component 1 (Com. 1, relative atomic mass), and component 2 (Com. 2, relative atomic mass). Here, macromolecular compounds are mainly referred to as nanoplastics. The difference between Com. 1 and Com. 2, for example, TiO_2_ (Com. 1 = 47.87, Com. 2 = 16.00) and graphene (Com. 1 = 12.01, Com. 2 = 0).
Shapes: granular NPs (Dim. 0, 0/1), one-dimensional NPs (Dim. 1, 0/1), two-dimensional NPs (Dim. 2, 0/1), and hollow (0/1).

Differences in plant species, NP properties, and growth conditions in the literature make direct comparisons of raw data difficult. The general use of the *z* score can lead to a serious reduction in model accuracy. To intuitively reflect the degree of the relative effects of different NPs on the plants under different experimental conditions, the label data (except RS ratio, TF, RCF and SCF) were converted to values between −1 and 1 based on the control and treated groups in the literature to reduce biases.

The formula is as follows:

[4] $$y^{\prime }=\left\{\begin{array}{ll} \frac{y-c}{y} & y>c\\ \frac{y-c}{c} & y<c \end{array}\right.$$

where *y* and *c* are the values of the experimental group and control group, respectively. $y^{\prime }$ is the converted value.

### Machine Learning Regression and Validation.

RF is a robust machine learning algorithm based on decision trees with strong anti-interference performance (63). RF has a strong ability to handle heterogeneous big data with quantitative and qualitative factors to provide solutions for predicting the fate of NPs in plants (22, 64). As a machine learning model integrating multiple decision trees, the RF model aggregates the results of each tree, performs classification analysis with a majority vote, and performs regression analysis with an average value. We used 20 variables that included NP properties, environmental factors, and experimental conditions as features (input data) for machine learning. The responses to NPs and uptake/transport in plants were used as labels (output data). RF models were built by the scikit-learn “RandomForestRegressor” in Python 3.8. The datasets were split into 13 subsets (3 growth subsets, 6 stress reaction subsets and 4 uptake and transport subsets as described above) based on different labels, and then 13 RF regression models were built, as listed in *SI Appendix*, Table S6. We adjusted two important parameters (*ntree* and *mtry*) of the RF model to optimize the predictive performance by the grid search method. *ntree* was set to 500, and *mtry* was set to 20. Approximately 36.8% of the raw data, which were called out-of-bag (OOB) data, were used to validate the model performance (63). OOB validation ensured that the RF was robust enough to avoid overfitting. The OOB percentage was calculated by the following equation:

[5] $$\lim_{n\to \infty }{\left(1-\frac{1}{n}\right)}^{n}=\frac{1}{e}\approx 0.368,$$

where *n* is the sampling frequency and *e* is the natural constant with a value of approximately 2.7183.

Moreover, we used the 10-fold cross-validation (ShuffleSplit) method for the 13 machine learning models to avoid overfitting. The dataset was randomly divided into 10 parts, nine of which were used as training sets and the rest as test sets to test the performance of the model. To measure the model performance, we averaged the *R*^2^ and RMSE values of the 10 regressions between the observations and predictions. TensorFlow Keras was used in Python 3.8 to build a two-layer connected ANN model. Similar to RF, the 10-fold cross-validation (ShuffleSplit) method was used to avoid overfitting in the ANN models. The hidden layer of the ANN model was set to 2*(*n*−1), where *n* is the number of features. Adam was used as the optimizer, and a small learning rate decay of 0.0001 was used to avoid overfitting. SVM models were built by “svm” in Python 3.8. Tenfold cross-validation (ShuffleSplit) was also applied. The kernel function used “rbf,” and 1 was set as the regularization parameter. XGB models were built by “XGBRegressor” in Python 3.8. Tenfold cross-validation (ShuffleSplit) was also applied. The *ntree* was set to 100.

### Overfitting Test.

We used a permutation test to represent the overfitting of the RF models. Random values randomly replaced 20, 40, 60, 80, and 100% of the label values in the training sets with the original label range in 10-fold cross-validation. Then, *Q*^2^ was calculated. The formula for *Q*^2^ is as follows (65):

[6] $$Q^{2}=1-\frac{\sum_{i=1}^{n}{\left(y_{i}-\hat{y}\right)}^{2}}{\sum_{i=1}^{n}{\left(y_{i}-\overset{-}{y}\right)}^{2}},$$

where $y_{i}$ is the observed label value, $\hat{y}$ is the predicted label value, and $\overset{-}{y}$ is the average value of the label. The permutation of each ratio was set to 20, 40, 60, 80, and 100%, and 500 permutation *Q*^2^ values were calculated for each model (5 ratios × 10 times/ratio × 10-fold). The calculated predicted *Q*^2^ values and the original labels were subjected to linear regression. If the intercept of the regression result on the *Y *axis is less than 0.05, the model is not overfitted (17).

### Feature Selection.

Feature selection for the model was performed using the SBS procedure. This method started with the full feature set, removed redundant features one by one, and found the optimal feature subset. The *R*^2^ of each selected subset was calculated to compare the performance of different feature combinations. We set the minimum number of features for the subsets (not less than half of the total number of features) to prevent the model from losing too much information. If there was no obvious improvement in the model performance through feature selection by the SBS algorithm, we chose the full features to build RF models for subsequent analysis to avoid missing important NP properties.

### Heterogeneous Distribution Visualization of NP Type and Shape.

A similarity network was used to visualize the heterogeneous distribution of priority factors in NP-acting plant models based on the Kamada–Kawai layout algorithm (66). The similarity matrix was obtained from the RF models through the Kamada–Kawai layout algorithm, and the “igraph” package in R 4.0.5 was used to draw the similarity network. Each data point in the model corresponded to a node in a similar network. The nodes were colored according to NP type or shape. The frequency of two data points appearing in the same node of a tree was used to quantify the factor–response dependence similarity in the proximity matrix. The value of two connected nodes was higher than a certain multiple (based on node number) of the average of the proximity matrix based on previous work (67). Data points with similarities in the RF model were reflected in the connected nodes. The cluster density was used to measure the tightness and heterogeneity of the network.

### Avoiding Bias Using Four Feature Importance Analyses.

Feature importance analysis of the RF models was performed using the “randomForest” and “randomForestExplainer” packages in R 4.0.5. MSE and node purity increases are widely used as measures of feature importance in machine learning. MSE and node purity increase because mathematical statistical indictors are affected by the quantity and quality of the datasets. Therefore, absolute dominant features may be incorrectly identified when a single importance evaluation index is used. To avoid the possible bias caused by a single importance criterion, we used a multiway feature importance analysis (increased MSE values, increased node purity, mean minimal depth, and *P* value) to screen important features. The MSE increase is a variable importance indicator based on the decrease in the predictive accuracy of the forest after perturbation of the variable. The MSE increase was computed by permuting OOB data. For each tree, the prediction error in the OOB portion of the data was recorded, with the error rate corresponding to the classification performance and the MSE indicating the regression performance. Then, the same procedure was performed after permuting each predictor variable. The differences between the initial MSE and MSE after permutation were then averaged over all trees and normalized by the SD of the differences. The variable that led to a larger MSE made a more important contribution to the output. The node purity increase indicated the total increase in node purity from splitting the variable averaged across all trees. Purity is the basis for determining the split of a decision tree node and is measured by the sum of the minimum mean square errors of the node. The variable that led to a higher node purity value made a more important contribution to the output. The mean minimal depth is a parameter from the structure of the forest and represents the distance of the feature to the tree root, where a closer root corresponds to a more important feature, and the *P* value indicates the significance of feature importance based on the one-sided binomial test. Partial dependence analysis was conducted using the “pdp” package for important features to obtain the relationship of the features to the plant responses and the uptake/transport of NPs.

### Feature Interaction and Covariance Analysis.

The number of occurrences in an RF model represents the strength of the interactions between two features (68). We analyzed the structure of each tree in the RF model and then obtained the number of feature-feature occurrences. We used the “randomForestExplainer” package in R 4.0.5 to calculate the number of occurrences between two features. The number of occurrences refers to the number of times that the two features appeared simultaneously on one tree of the forest as the nodes were split. A larger number of occurrences represents a stronger interaction between the two features in the RF model. Subsequently, we selected the feature–feature with the top 50 occurrence frequencies and established the feature interaction network. Network graphs obtained with the Gephi 0.9.2 software package were used to visualize the strength of the feature-to-feature interactions in the models.

Based on the frequency of NPs and plants in the literature, we selected specific NPs and plants for two-feature covariance analysis (double-variable partial dependence analysis). Two features of the NP properties and experimental conditions with the strongest interaction were screened out for covariance analysis (e.g., total NP exposure dose-duration and zeta potential- size_TEM_). In the study of the plant dry weight response, the total NP exposure dose ranged from 0 to 50 mg in 100 steps of 0.5 mg; the duration ranged from 0 to 50 d in 100 steps of 0.5 d; the zeta potential of the NPs ranged from −50 to 0 mV in 100 steps of 0.5 mV; and the size_TEM_ of the NPs ranged from 0 to 100 nm in 100 steps of 1 nm. In the study of the plant superoxide dismutase response, the NP total exposure dose ranged from 0 to 50 mg in 100 steps of 0.5 mg; the duration ranged from 0 to 50 d in 100 steps of 0.5 d; the zeta potential of the NPs ranged from −50 to 50 mV in 100 steps of 1 mV; and the size_TEM_ of the NPs ranged from 0 to 100 nm in 100 steps of 1 nm. In the study of the crop chlorophyll *b* response, the total NP exposure dose ranged from 0 to 50 mg in 100 steps of 0.5 mg; the duration ranged from 0 to 50 days in 100 steps of 0.5 d; the zeta potential of the NPs ranged from −50 to 50 mV in 100 steps of 1 mV; and the size_TEM_ of the NPs ranged from 0 to 100 nm in 100 steps of 1 nm. In the study of the crop uptake response, the total NP exposure dose ranged from 0 to 50 mg in 100 steps of 0.5 mg; the duration ranged from 0 to 50 d in 100 steps of 0.5 d; the zeta potential of the NPs ranged from −50 to 50 mV in 100 steps of 1 mV; and the size_TEM_ of the NPs ranged from 0 to 100 nm in 100 steps of 1 nm. For each analysis, we ran each model 10,000 times. The above feature range settings were based on the value distributions of the features in the datasets.

### Global Predictions.

The responses of global crops (bean, wheat, and maize) and the uptake of NPs in the first 21 d (seedling stage) of the real planting stage in 2018 (the most current and intact datasets we could obtain) were predicted using the RF models at a 5-arcmin spatial resolution. The seedling period of plants is relatively short and sensitive to NPs. The planting time of global crops is fixed, providing certain time parameters and field parameters for prediction. Considering the frequency and cost of application in agriculture, we use the most common ZnO and Fe_2_O_3_ NPs for global prediction instead of precious metals. Partial dependence analysis showed that the plant response and uptake of NPs changed with the total NP exposure dose (*SI Appendix*, Fig. S16). In *SI Appendix*, Fig. S16*A*, with the increase in the total exposure dose at 20 to 80 mg, the influence on plant length tended to change slightly, and we set the total exposure dose to 50 mg for further plant length studies. With the increase in the exposure dose to 50 mg, the oxidative stress index and the uptake of NP by plants tended to be stable (*SI Appendix*, Fig. S16 *B* and *D*), and 50 mg was used for further oxidative stress and uptake studies. The input data included specific NPs and crop properties, as well as the field global gridded dataset of daytime and night temperatures (data source, https://neo.gsfc.nasa.gov/), soil types (data source, https://www.soilgrids.org/), illumination intensity (data source, https://neo.gsfc.nasa.gov/), and relative humidity (data source, https://cds.climate.copernicus.eu/) (*SI Appendix*, Tables S3–S5). The resolution of the field data was unified to 5 arcmin, and grids with missing data were deleted. Daytime and night temperatures, illumination intensity, and relative humidity over the growing season for a given crop type were acquired from the corresponding dataset, where the planting season in each grid cell was identified as the planting dates obtained from the literature (44). Daytime and night temperature data were based on the monthly average temperature on the planting dates, as well as the illumination intensity and relative humidity. The soil datasets were classified into acid, neutral, and alkaline soil types based on the pH value (6.5 > pH, acid; 6.5 ≤ pH ≤ 7.5, neutral, and 7.5 < pH, alkaline). To apply the established machine learning model to actual field prediction, field data (e.g., the global gridded dataset of daytime and night temperatures, soil types, illumination, and relative humidity) were employed in the models. The data from laboratory experiments (e.g., daytime and night temperatures, soil types, illumination intensity, and relative humidity) have one-to-one correspondence with the field features in the models. The five feature ranges in the laboratory dataset almost cover the field features. Therefore, the model established based on laboratory and field data can project the situation. Predicted values of root length and NP uptake were mapped directly on the map using the ArcGIS 10.6 software package. Due to the lack of a composite indicator of oxidative stress, we used the absolute value of the average variation of the four oxidative indicators (ascorbate peroxidase, hydrogen peroxide, malondialdehyde, and superoxide dismutase) to represent the oxidative stress of the plants. Normalized absolute values (maximum value was 1) were sorted and divided into 20 equal parts representing the relative levels of oxidative stress. Higher levels represent greater potential oxidative stress. The machine learning model is data-driven, and the differences in spatial climate data will generate different spatial responses. Climate data and the Shapley value explained the differences in the spatial responses. The Shapley value can be used for local interpretation of the model and reflects the contribution of features (69, 70). Based on the RF model, “shap” was used to calculate the Shapley values of the environmental factors (daytime and night temperatures, illumination intensity, and relative humidity) of each cell in Python 3.8. The factor with the higher Shapley value acted as the more important factor.

To predict plant length, oxidative stress, and NP uptake under future climate conditions, future meteorological field data were obtained from ACCESS-CM_2_ in the Coupled Model Intercomparison Project, now in its sixth phase (CMIP6) (https://www.worldclim.org/). CMIP6 climate models from the World Climate Research Programme (WRCP) play an important role in supporting climate research and are widely used for the prediction of future crop yield, although there is uncertainty from different coupling modes of climate models (71). Based on the CMIP6 climate model, a reduction in wheat yield was predicted in France under future climate change (72), and the impact of different scenarios on global maize, soybean, and rice yields were also analyzed (73). The above research indicates the feasibility and reliability of combining the CMIP6 climate model with crop prediction and provides solutions for the global study of NPs with plants under different temperature increase scenarios. In this study, we focused on the effects of temperature changes on future meteorological data. The difference between the annual mean temperatures in 2018 and 2080 to 2100 (2090s) was calculated, and then the difference values were superimposed with the daytime and night temperatures in 2018 to obtain the daytime and night temperatures in the 2090s. In total, four future scenarios were selected (74), i.e., the sustainable scenario (SSP126), medium forcing scenario (SSP245), medium to high forcing scenario (SSP370), and high forcing scenario (SSP585).

### Uncertainty Analysis.

For global prediction models, the uncertainty was associated with differences in laboratory and field conditions as well as the quality of input data. To assess the uncertainty related to the global soil and climate data and unexplained variability not captured by the RF model, we randomly ran the RF model 200 times. Maps of the global distribution of root length, NP uptake and oxidative stress were built based on the mean values of the model prediction results. The corresponding map of the relative uncertainty of prediction was built based on the SD divided by the predicted mean.

## Supplementary Material

Appendix 01 (PDF)

Dataset S01 (XLSX)

## Data Availability

All datasets are available at https://github.com/dp1999nku/All-datasets (75). Code in the paper is available at https://github.com/dp1999nku/Code-supplement (76).
