# Supplementary material for: Development potential of nanoenabled agriculture projected using machine learning
Source: Proc Natl Acad Sci U S A. 2023 Jun 14;120(25):e2301885120. doi: 10.1073/pnas.2301885120 (PMC10288598; doi:10.1073/pnas.2301885120)
Supplement: Supplementary file 1 — Appendix 01 (PDF) [file pnas.2301885120.sapp.pdf]

Supplementary information

Development potential of nanoenabled agriculture projected using machine learning

Peng Deng[1], Yiming Gao[1], Li Mu[2, *], Xiangang Hu[1, *], Fubo Yu[1], Yuying Jia[1], Zhenyu Wang[3], Baoshan Xing[4]

[1]Key Laboratory of Pollution Processes and Environmental Criteria (Ministry of Education), Tianjin Key Laboratory of Environmental Remediation and Pollution Control, College of Environmental Science and Engineering, Nankai University, Tianjin 300350, China.

[2]Key Laboratory for Environmental Factors Controlling Agro-Product Quality Safety (Ministry of Agriculture and Rural Affairs), Tianjin Key Laboratory of Agro-Environment and Product Safety, Institute of Agro-Environmental Protection, Ministry of Agriculture and Rural Affairs, 300191, Tianjin, China.

[3]Institute of Environmental Processes and Pollution Control, School of Environment and Civil Engineering, Jiangnan University, Wuxi, Jiangsu 214122, China.

[4]Stockbridge School of Agriculture, University of Massachusetts, Amherst, MA 01003, United States

Corresponding authors:
Xiangang Hu; Email: huxiangang@nankai.edu.cn
Li Mu; Email: muli@caas.cn

Supplementary figures

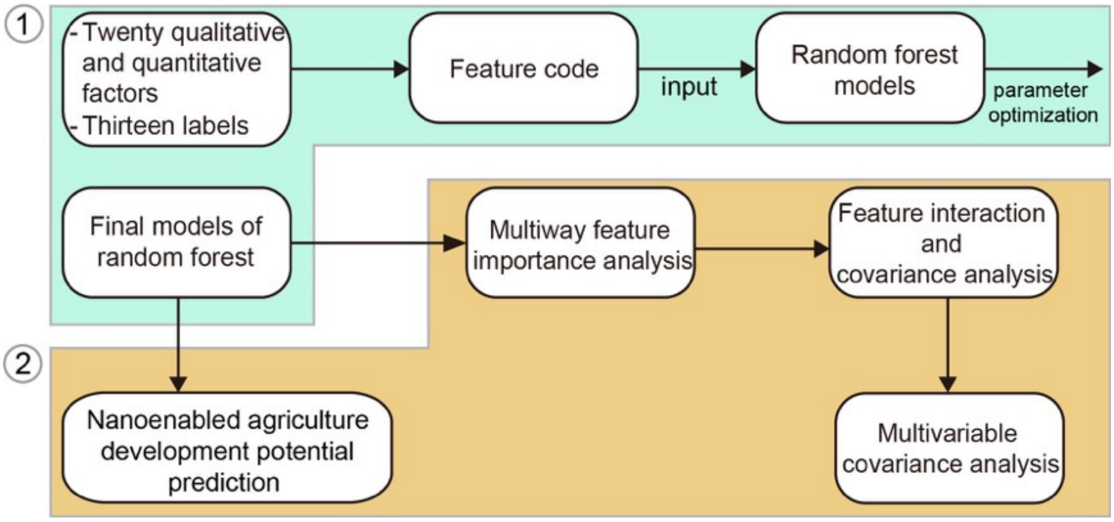

Fig. S1. Flow chart. ① Process of building a random forest model. ② Analysis based on the random forest model.

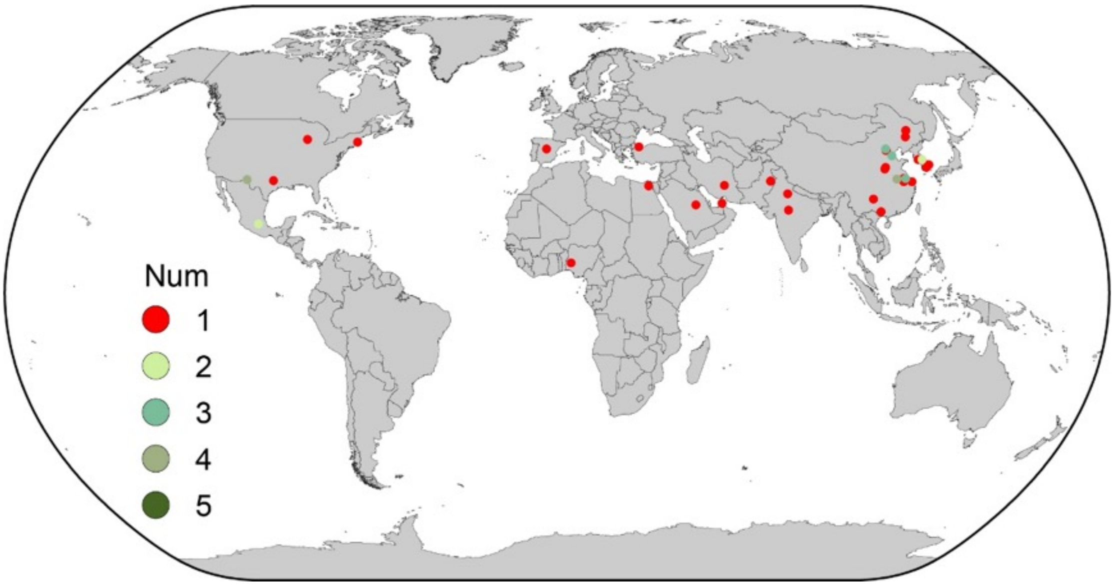

Fig. S2. Global geographical distribution of samples. The colours of the dots represent numbers. A total of 1174 datasets were collected from 57 publications.

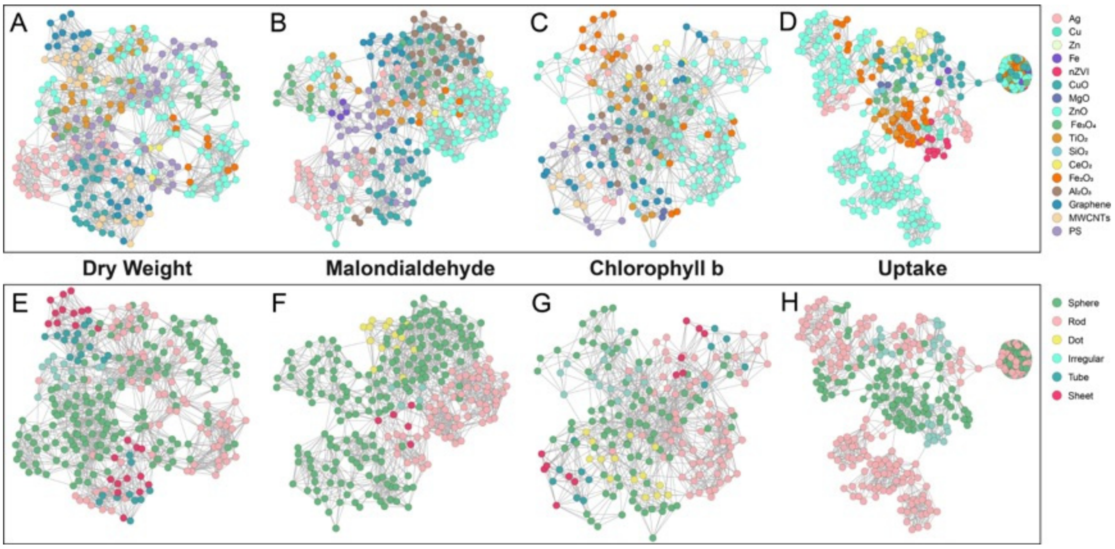

Fig. S3. The similarity network visualizes the data heterogeneity of plants with NPs. Each node represents a data point. NP type (A-D) and NP shape (E-H). The multiples of the values connected by nodes are greater than the average value of the proximity matrix and are 3, 5, 4 and 3 for dry weight, malondialdehyde, chlorophyll *b* and uptake, respectively. Tight connections in the similarity network indicate that the clusters are highly homogeneous and *vice versa*.

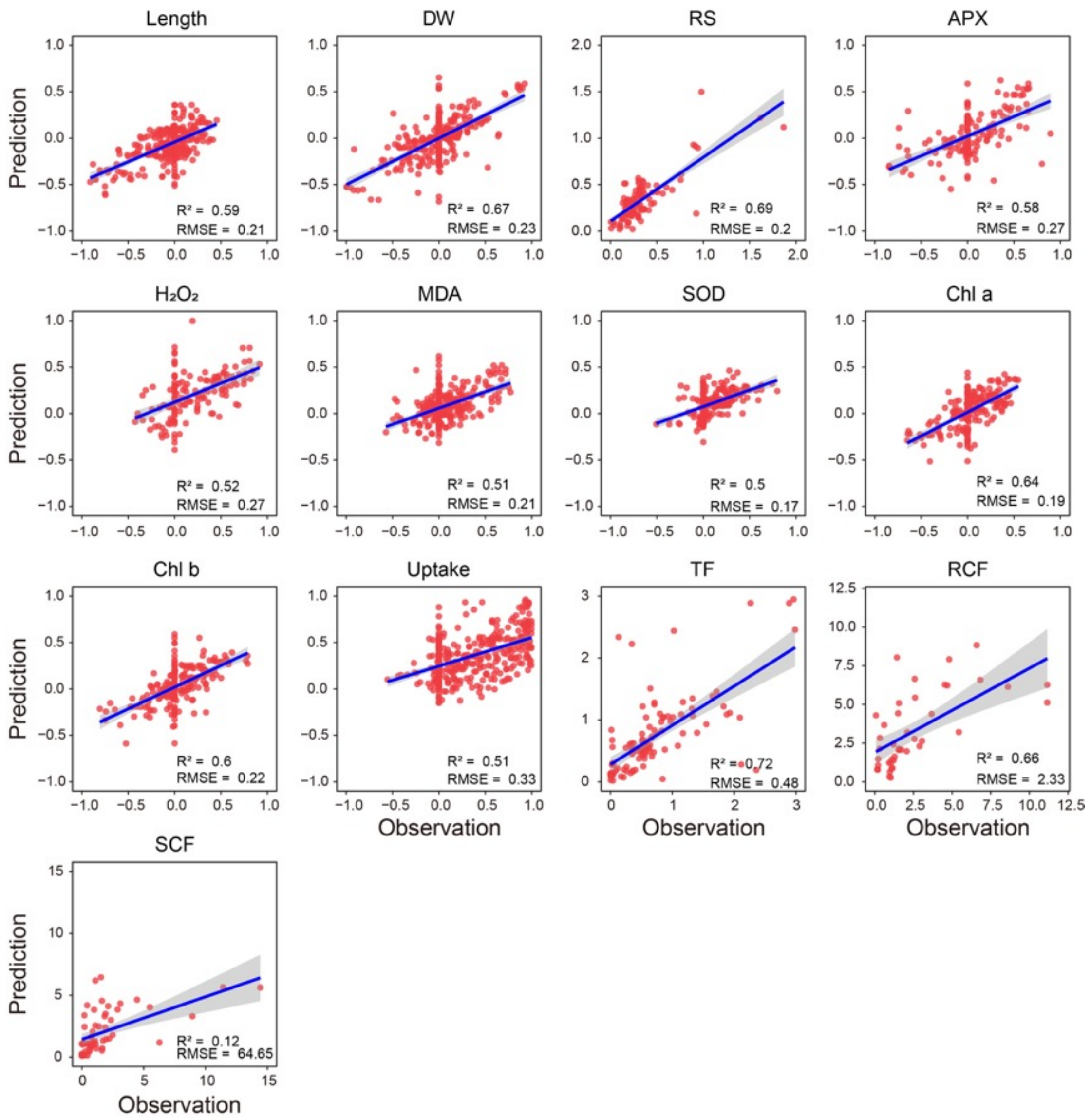

Fig. S4. Performance of multiple linear regression for heterogeneous data. The X-axis represents the observed values of the samples (measured data), and the Y-axis represents the predicted values. DW, dry weight; RS, root-shoot ratio; APX, ascorbate peroxidase; MDA, malondialdehyde; SOD, superoxide dismutase; Chl a, chlorophyll *a*; Chl b, chlorophyll *b*; TF, transport factor; RCF, root concentration factor; SCF, shoot concentration factor.

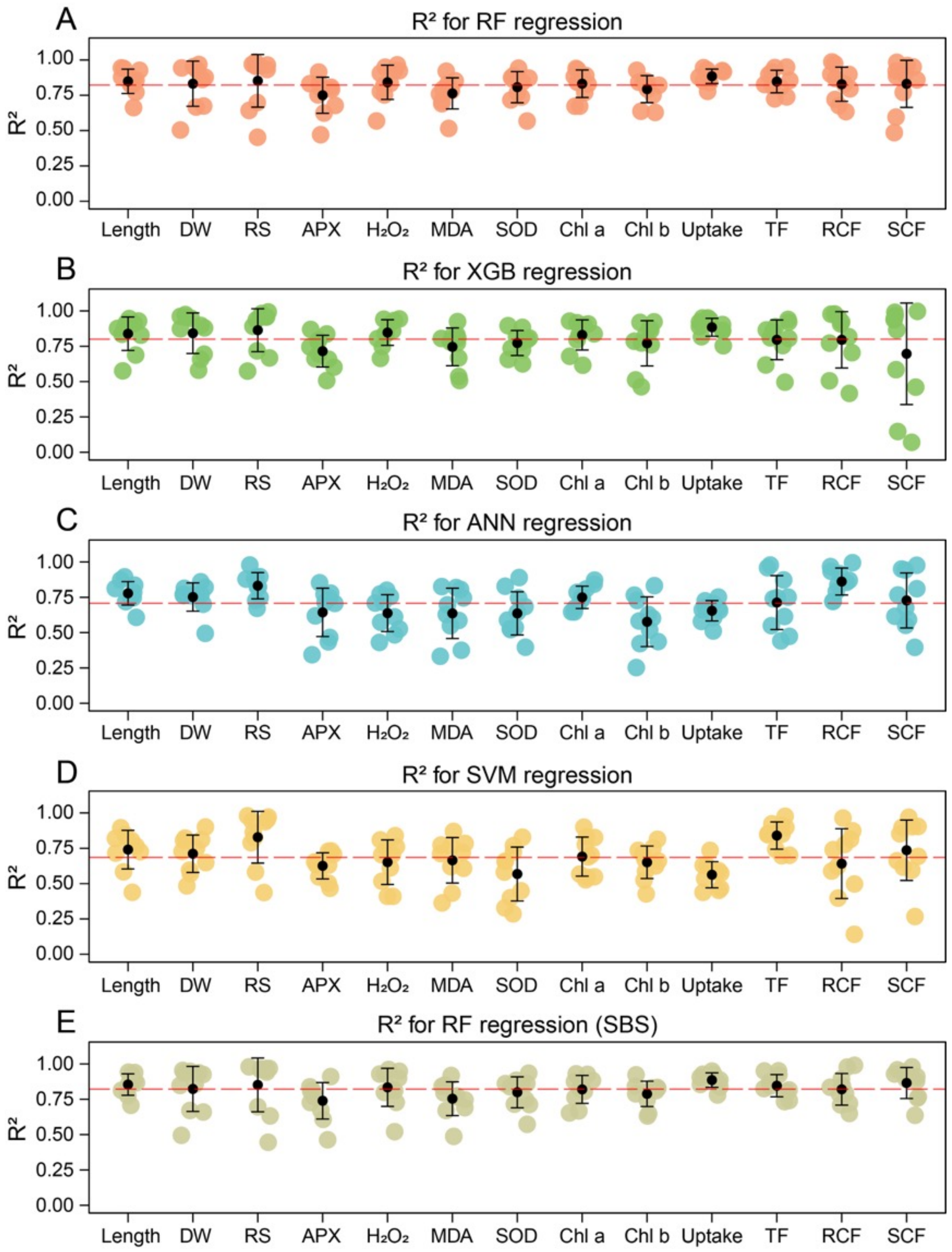

Fig. S5. Performance of machine learning models. (A) Random forest model; (B) extreme gradient boosting model; (C) artificial neural network model; (D) support vector machine model and (E) random forest model with sequential backwards selection (SBS) feature selection.

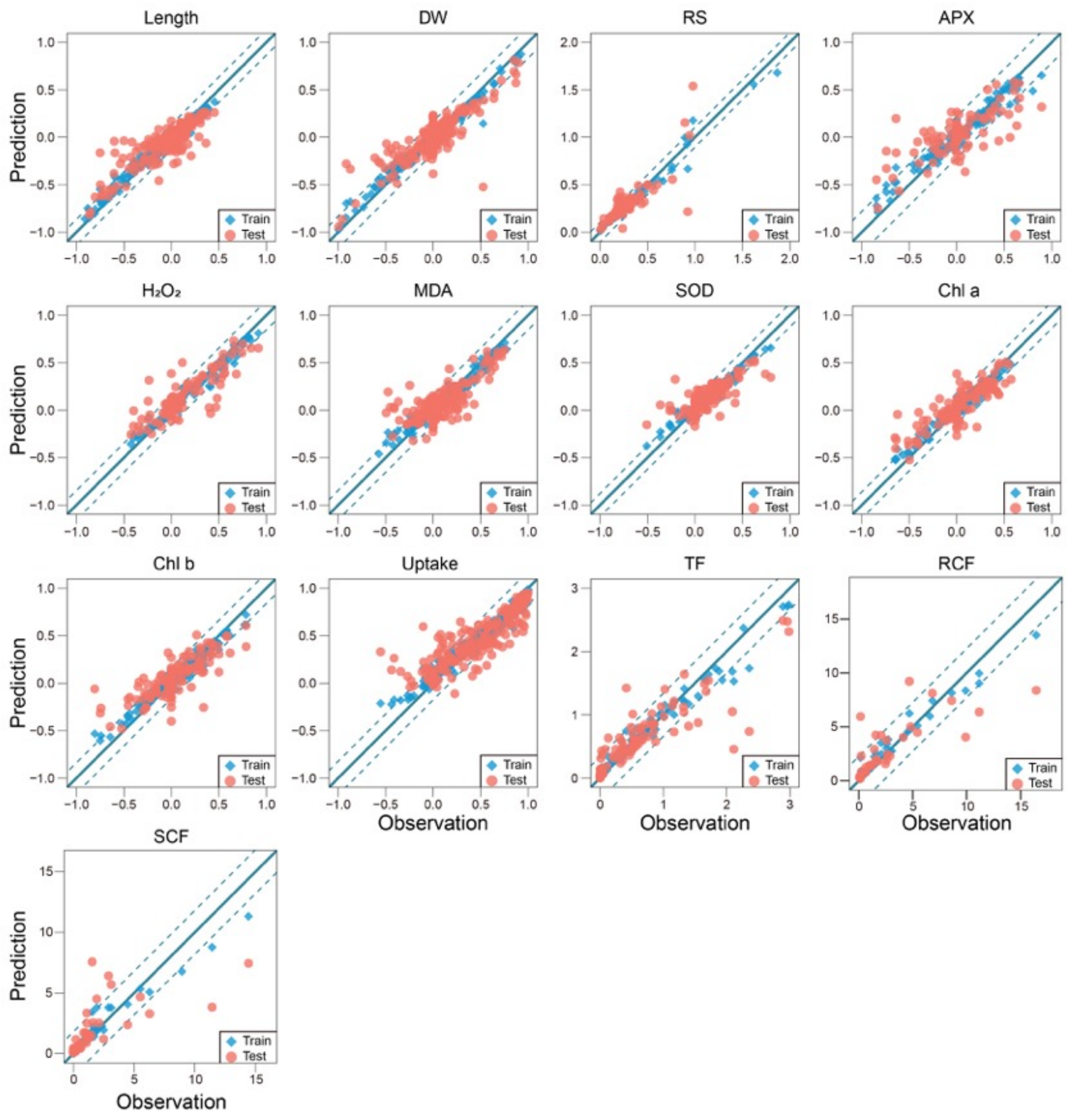

Fig. S6. Performance of the RF models in the prediction of plant responses and NP uptake/transport. The slope of the solid lines is 1, and the dotted lines represent the slopes of ±RMSE.

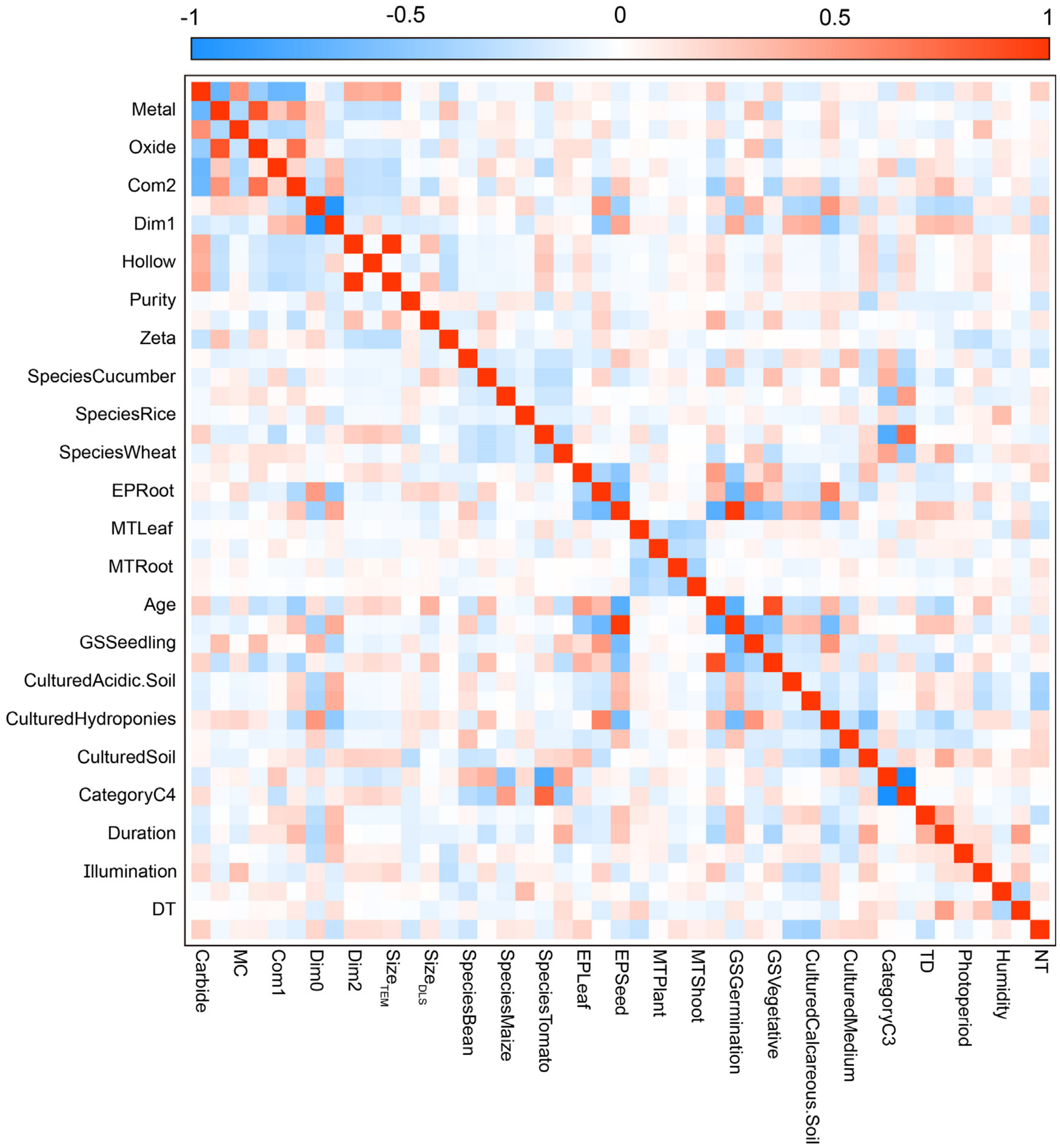

Fig. S7. Pearson correlation coefficient matrix of the variables in datasets. MC, macromolecular compound; Com. 1, component 1; Com. 2, component 2; Dim0, granular; Dim1, one-dimensional; Dim2, two-dimensional; EP, exposure pathway; MT, measured tissue; GS, growing stage; DT, daytime temperature; NT, night temperature.

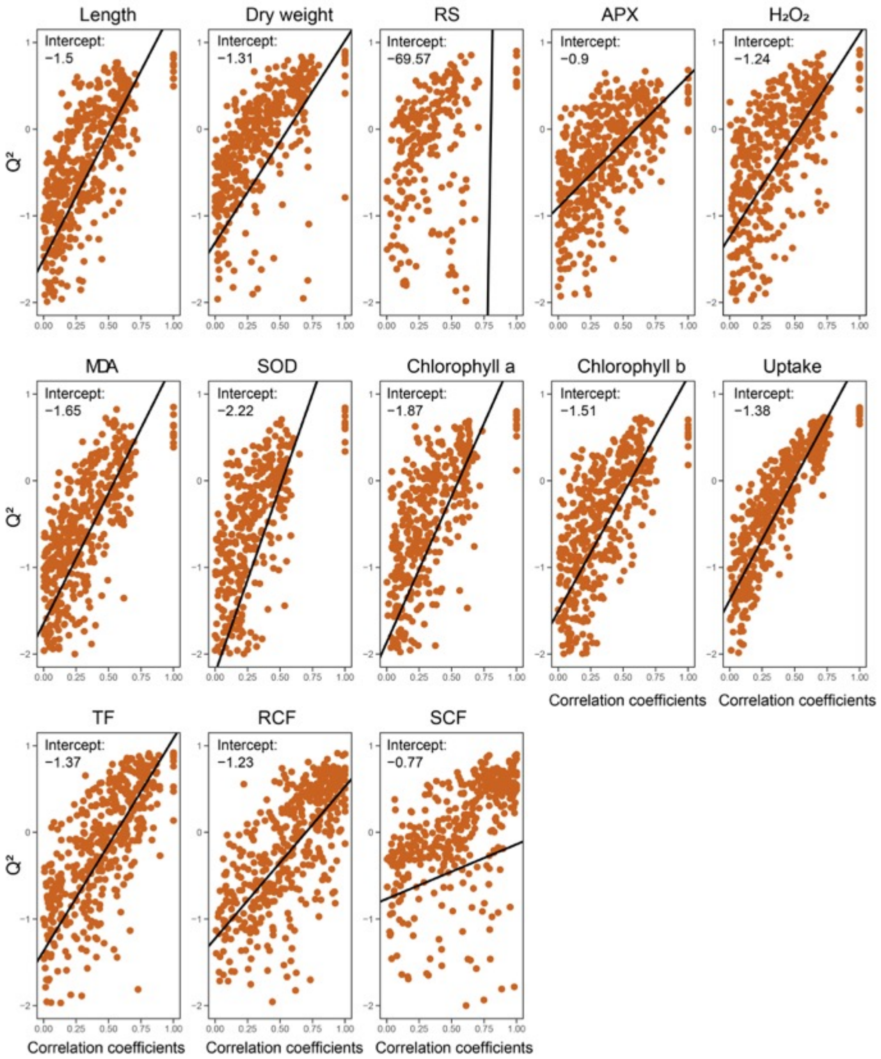

Fig. S8. Permutation test for overfitting. The X-axis represents the correlation coefficients of the raw label values and permutated label values, and the Y-axis represents the Q^2 values. The permutation test results show that none of the models overfit (intercept < 0.05). APX, ascorbate peroxidase; H_2O_2, hydrogen peroxide; MDA, malondialdehyde; SOD, superoxide dismutase.

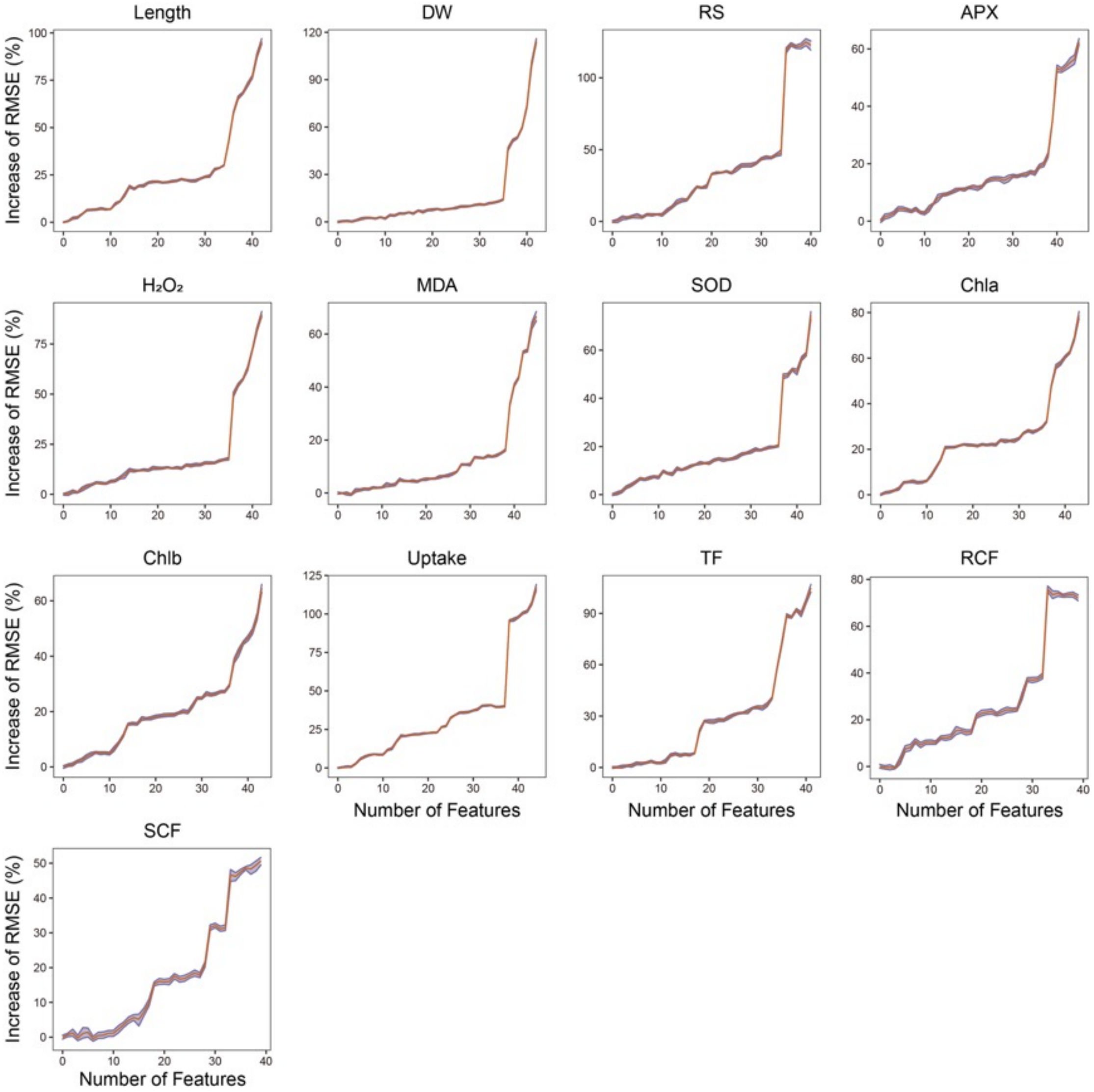

Fig. S9. Feature value shuffling. For all of the models, predictive performance was degraded after feature value shuffling, ensuring that the features contributed valid information to the models.

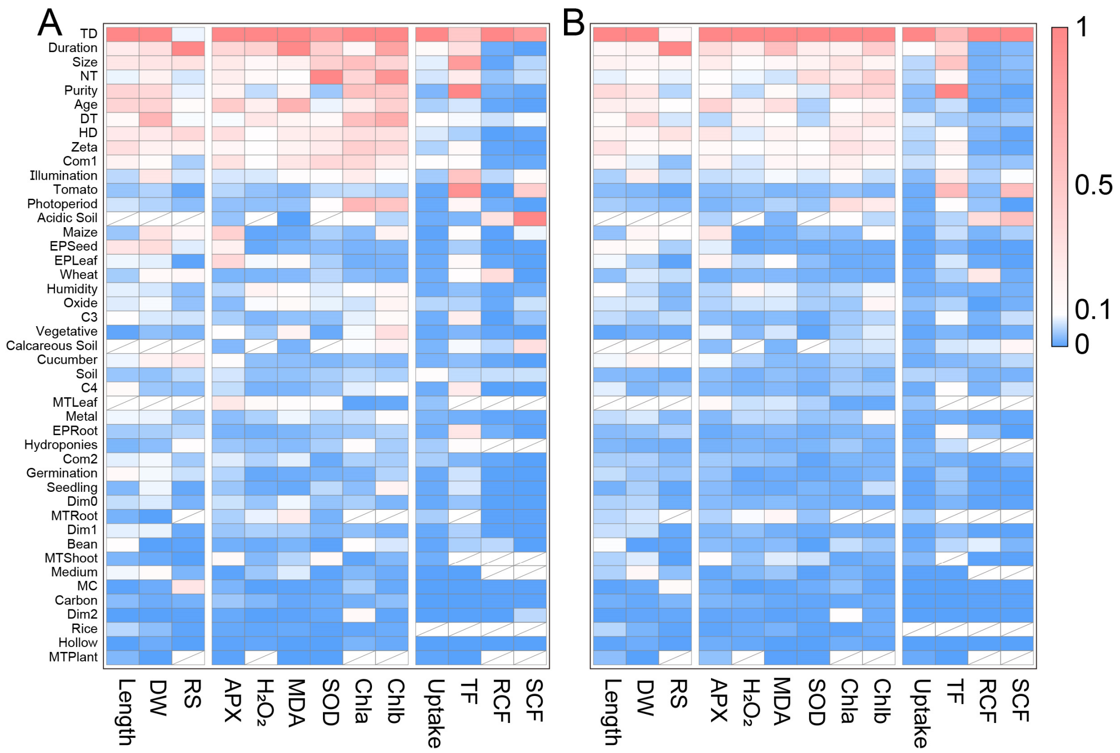

Fig. S10. Feature importance analysis of the plant response and NP uptake/transport. The normalized percentages of the MSE increase (A) and the normalized node purity increase (B) were employed to measure the feature importance.

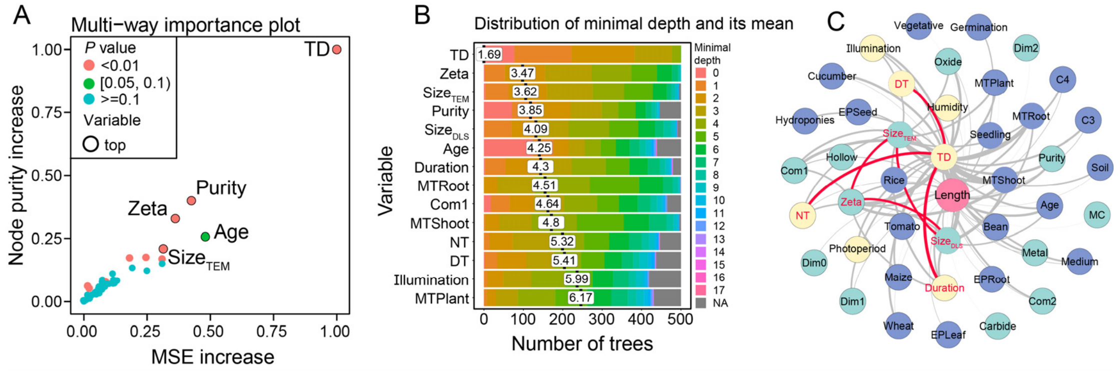

Fig. S11. Feature importance and interaction analysis. (A) Multiway feature importance analysis of the plant length model combining the MSE increase, node purity increase, and *P* values of the features. (B) Distribution of the features' mean minimal depth. (C) Feature interaction network of plant length based on the number of occurrences. Nodes of different colours represent different types of features and indicators. The line between the two features represents the top 50 interaction strengths based on the number of occurrences. The three strongest NP properties and experimental condition interactions in the networks are highlighted in red.

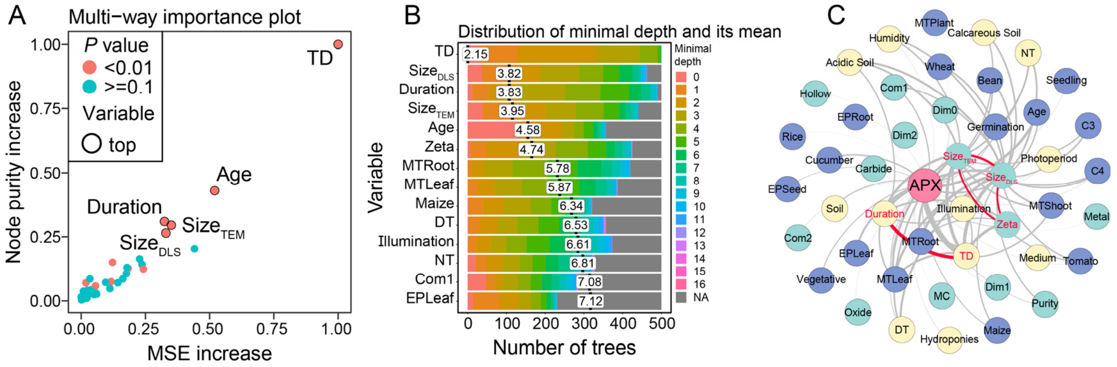

Fig. S12. Feature importance and interaction analysis. (A) Multiway feature importance analysis of the ascorbate peroxidase model combining the MSE increase, node purity increase, and *P* values of the features. (B) Distribution of the features' mean minimal depth. (C) Feature interaction network of ascorbate peroxidase based on the number of occurrences. Nodes of different colours represent different types of features and indicators. The line between the two features represents the top 50 interaction strengths based on the number of occurrences. The three strongest NP properties and experimental condition interactions in the networks are highlighted in red.

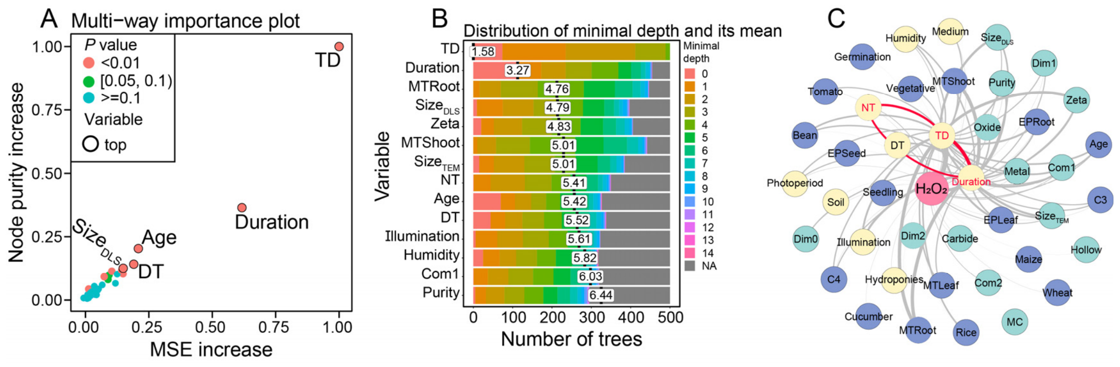

Fig. S13. Feature importance and interaction analysis. (A) Multiway feature importance analysis of the hydrogen peroxide model combining the MSE increase, node purity increase, and *P* values of the features. (B) Distribution of the features' mean minimal depth. (C) Feature interaction network of hydrogen peroxide based on the number of occurrences. Nodes of different colours represent different types of features and indicators. The line between the two features represents the top 50 interaction strengths based on the number of occurrences. The three strongest NP properties and experimental condition interactions in the networks are highlighted in red.

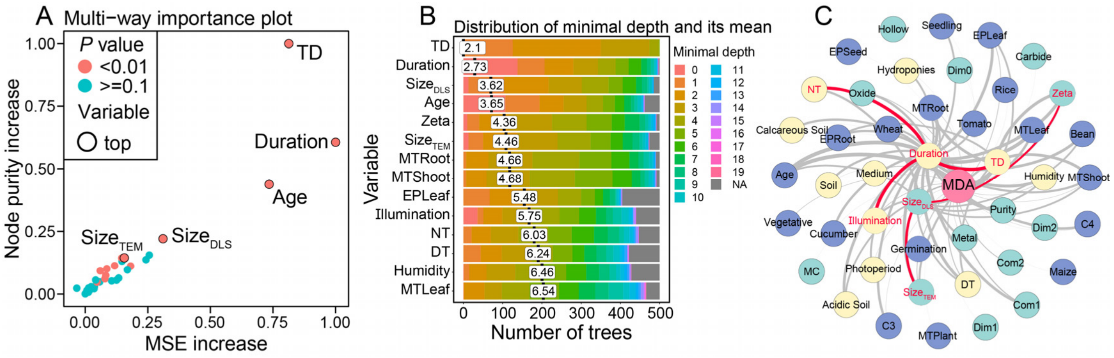

Fig. S14. Feature importance and interaction analysis. (A) Multiway feature importance analysis of the malondialdehyde model combining the MSE increase, node purity increase, and *P* values of the features. (B) Distribution of the features' mean minimal depth. (C) Feature interaction network of malondialdehyde based on the number of occurrences. Nodes of different colours represent different types of features and indicators. The line between the two features represents the top 50 interaction strengths based on the number of occurrences. The three strongest NP properties and experimental condition interactions in the networks are highlighted in red.

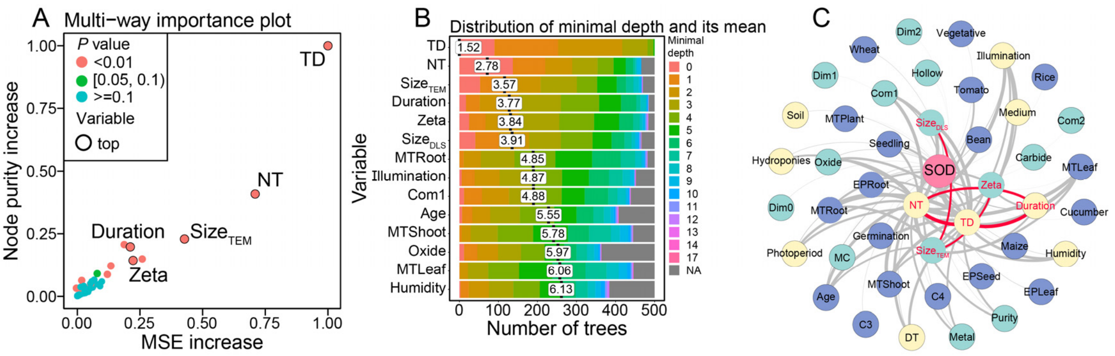

Fig. S15. Feature importance and interaction analysis. (A) Multiway feature importance analysis of the superoxide dismutase model combining the MSE increase, node purity increase, and *P* values of the features. (B) Distribution of the features' mean minimal depth. (C) Feature interaction network of superoxide dismutase based on the number of occurrences. Nodes of different colours represent different types of features and indicators. The line between the two features represents the top 50 interaction strengths based on the number of occurrences. The three strongest NP properties and experimental condition interactions in the networks are highlighted in red.

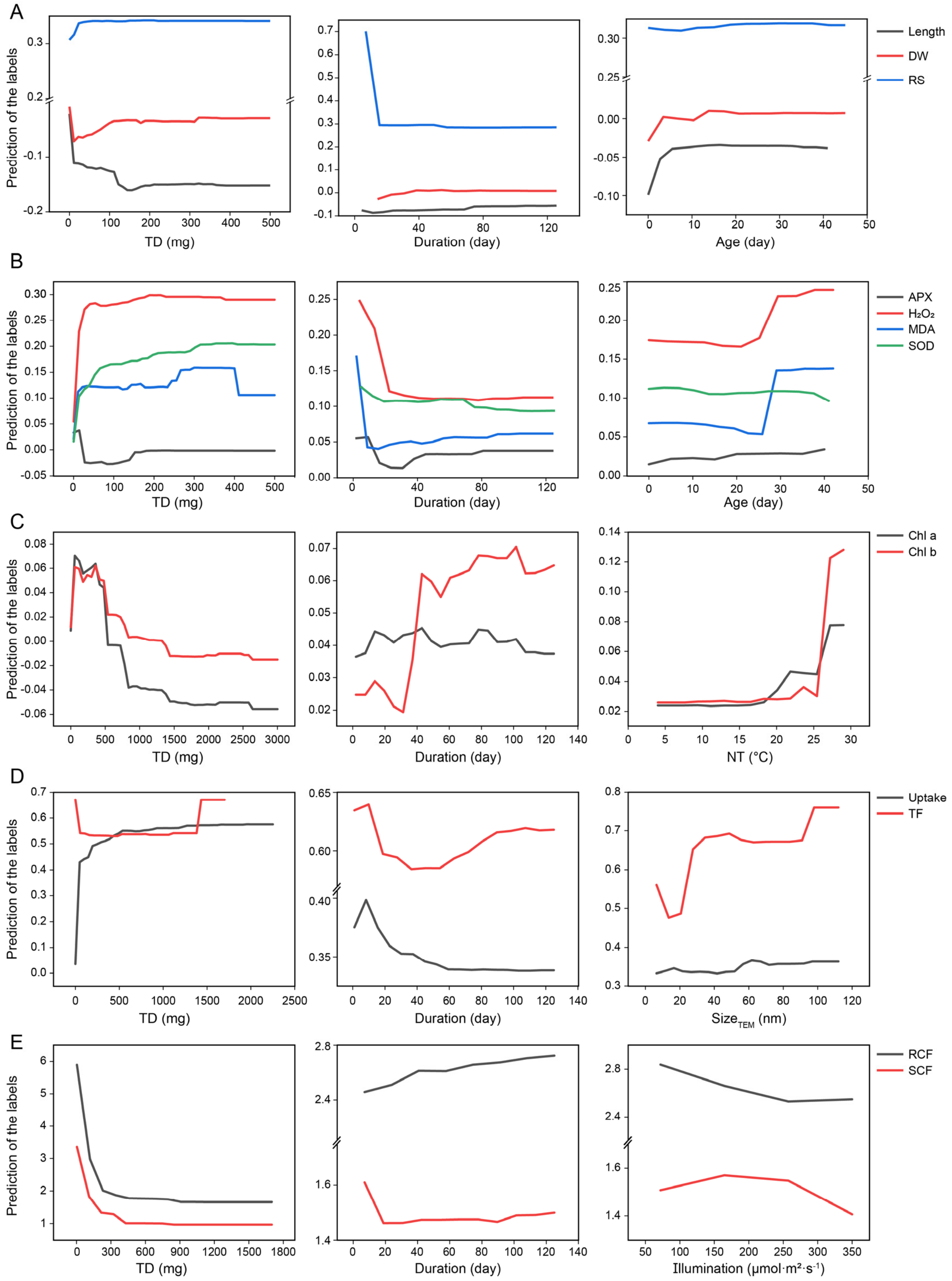

Fig. S16. Partial dependence analysis of important features. (A) Growth response; (B) oxidative stress response; (C) photosynthesis and (D) NP uptake/transport.

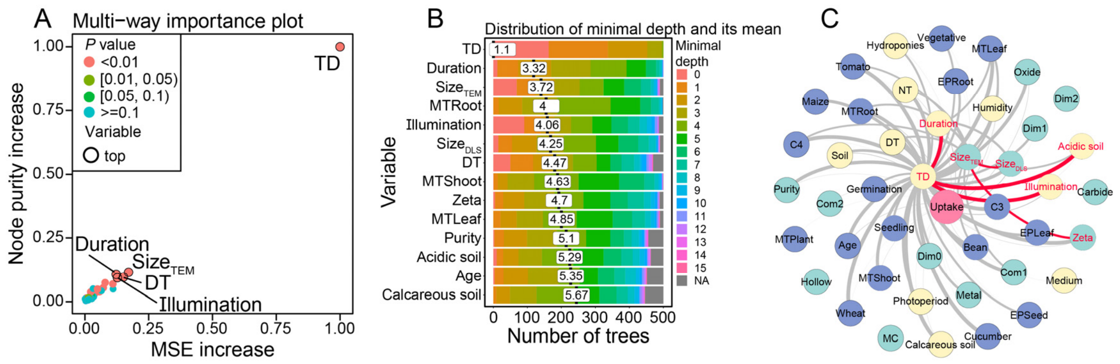

Fig. S17. Feature importance and interaction analysis. (A) Multiway feature importance analysis of the uptake model combining the MSE increase, node purity increase, and *P* values of the features. (B) Distribution of the features' mean minimal depth. (C) Feature interaction network of uptake based on the number of occurrences. Nodes of different colours represent different types of features and indicators. The line between the two features represents the top 50 interaction strengths based on the number of occurrences. The three strongest NP properties and experimental condition interactions in the networks are highlighted in red.

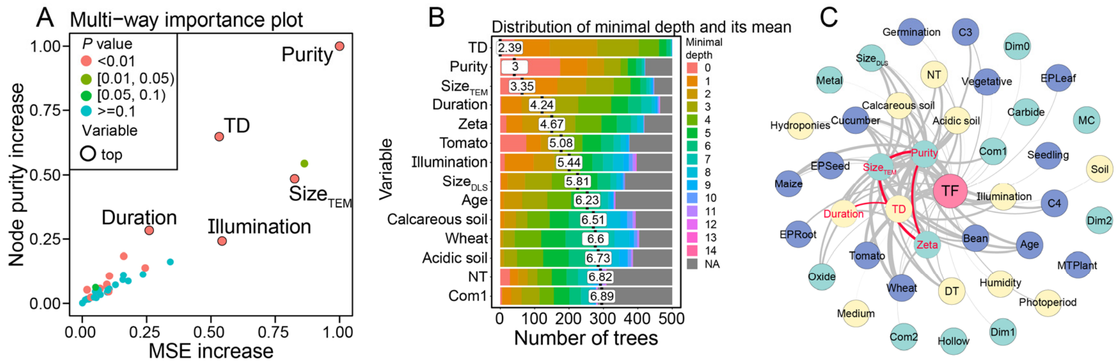

Fig. S18. Feature importance and interaction analysis. (A) Multiway feature importance analysis of the transport factor model combining the MSE increase, node purity increase, and *P* values of the features. (B) Distribution of the features' mean minimal depth. (C) Feature interaction network of the transport factor based on the number of occurrences. Nodes of different colours represent different types of features and indicators. The line between the two features represents the top 50 interaction strengths based on the number of occurrences. The three strongest NP properties and experimental condition interactions in the networks are highlighted in red.

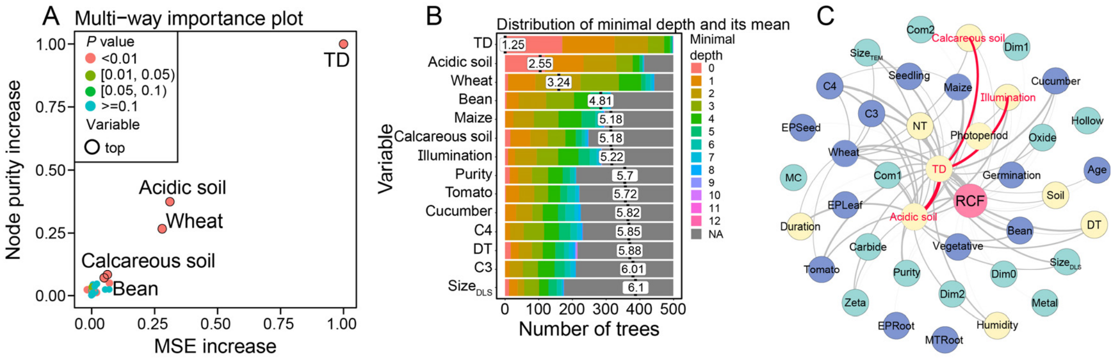

Fig. S19. Feature importance and interaction analysis. (A) Multiway feature importance analysis of the root concentration factor model combining the MSE increase, node purity increase, and *P* values of the features. (B) Distribution of the features' mean minimal depth. (C) Feature interaction network of the root concentration factor based on the number of occurrences. Nodes of different colours represent different types of features and indicators. The line between the two features represents the top 50 interaction strengths based on the number of occurrences. The three strongest NP properties and experimental condition interactions in the networks are highlighted in red.

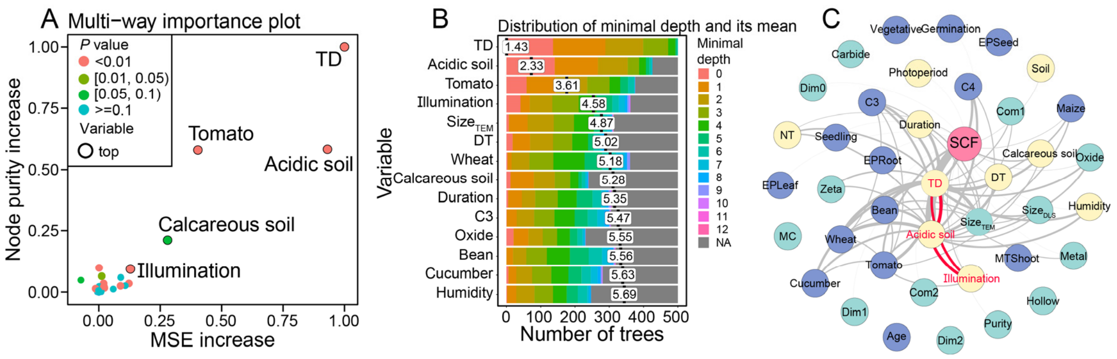

Fig. S20. Feature importance and interaction analysis. (A) Multiway feature importance analysis of the shoot concentration factor model combining the MSE increase, node purity increase, and *P* values of the features. (B) Distribution of the features' mean minimal depth. (C) Feature interaction network of the shoot concentration factor based on the number of occurrences. Nodes of different colours represent different types of features and indicators. The line between the two features represents the top 50 interaction strengths based on the number of occurrences. The three strongest NP properties and experimental condition interactions in the networks are highlighted in red.

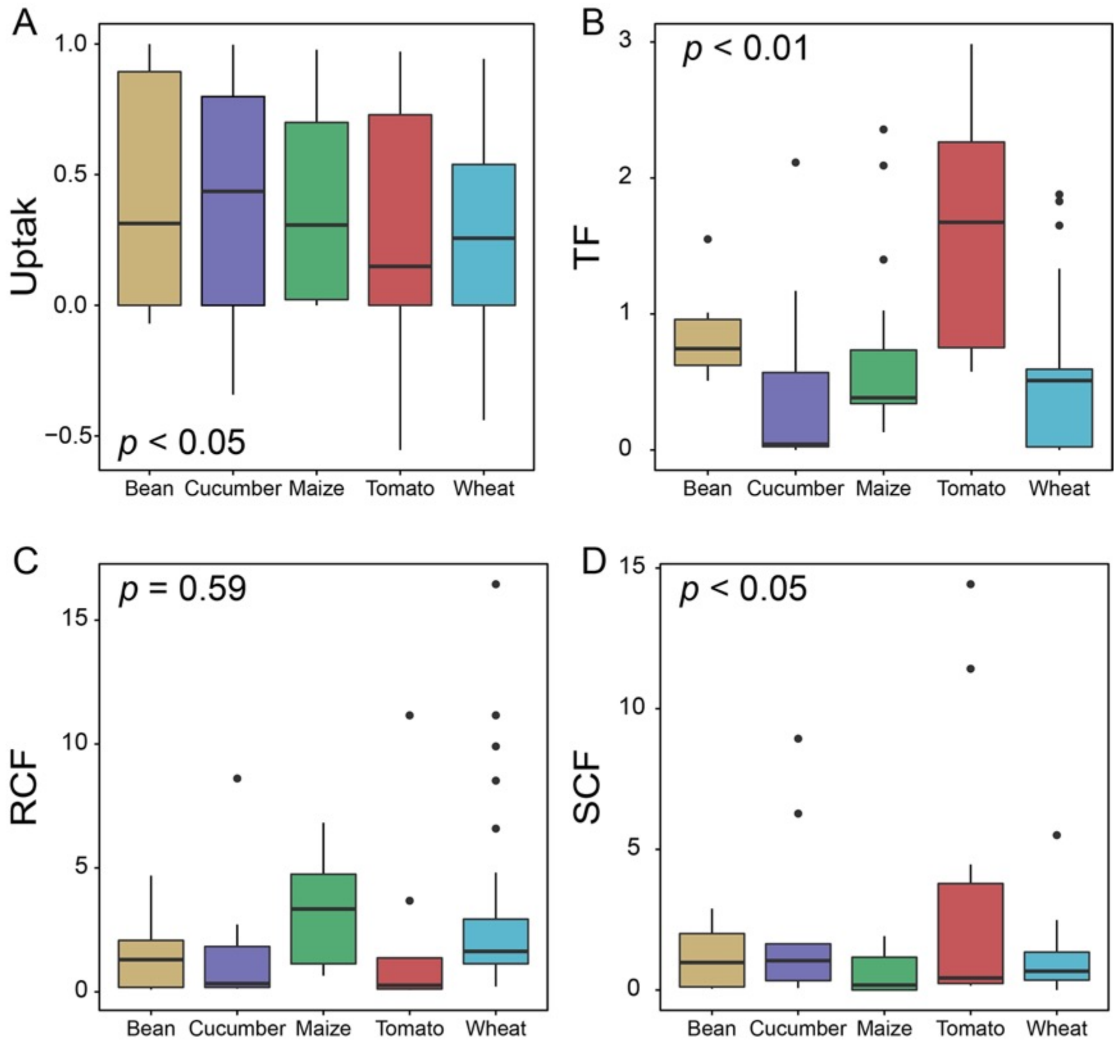

Fig. S21. Data distribution of NP uptake and transport in five different plant species. (A) Uptake; (B) transport factor (TF); (C) root concentration factor (RCF) and (D) shoot concentration factor (SCF). One-way ANOVA was used for significant differences analysis among five different plant types. A *p* value < 0.05 was considered to indicate statistical significance.

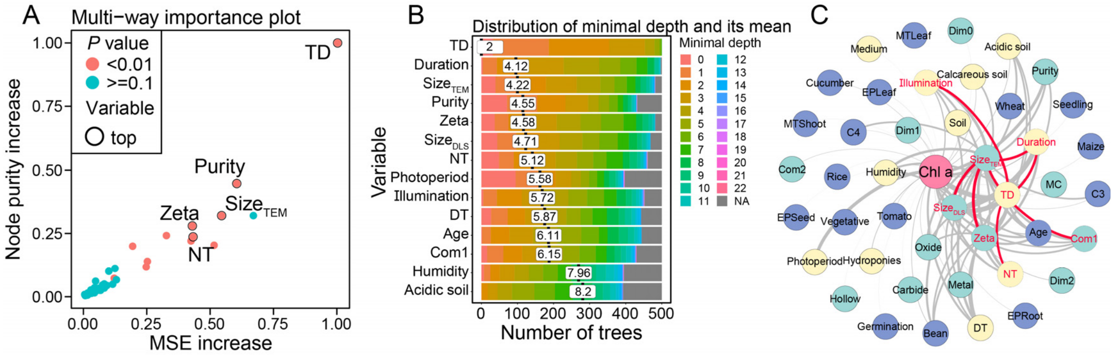

Fig. S22. Feature importance and interaction analysis. (A) Multiway feature importance analysis of the chlorophyll *a* model combining the MSE increase, node purity increase, and *P* values of the features. (B) Distribution of the features' mean minimal depth. (C) Feature interaction network of chlorophyll a based on the number of occurrences. Nodes of different colours represent different types of features and indicators. The line between the two features represents the top 50 interaction strengths based on the number of occurrences. The three strongest NP properties and experimental condition interactions in the networks are highlighted in red.

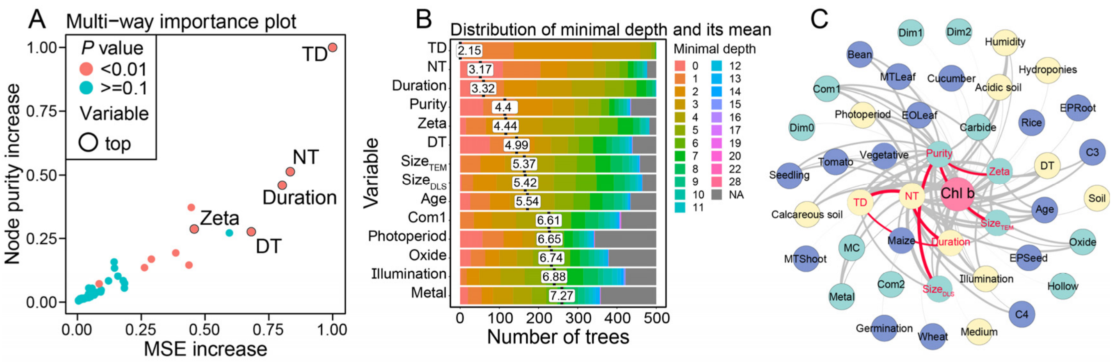

Fig. S23. Feature importance and interaction analysis. (A) Multiway feature importance analysis of the chlorophyll *b* model combining the MSE increase, node purity increase, and *P* values of the features. (B) Distribution of the features' mean minimal depth. (C) Feature interaction network of chlorophyll b based on the number of occurrences. Nodes of different colours represent different types of features and indicators. The line between the two features represents the top 50 interaction strengths based on the number of occurrences. The three strongest NP properties and experimental condition interactions in the networks are highlighted in red.

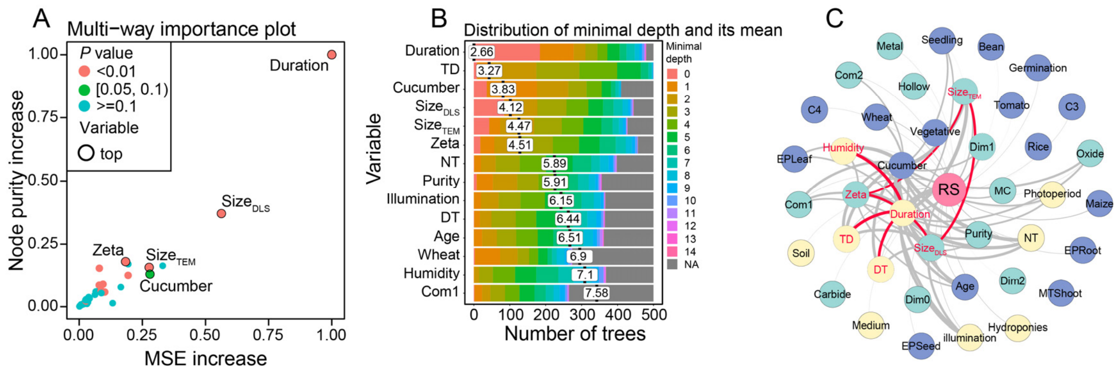

Fig. S24. Feature importance and interaction analysis. (A) Multiway feature importance analysis of the root/shoot ratio model combining the MSE increase, node purity increase, and *P* values of the features. (B) Distribution of the features' mean minimal depth. (C) Feature interaction network of the root/shoot ratio based on the number of occurrences. Nodes of different colours represent different types of features and indicators. The line between the two features represents the top 50 interaction strengths based on the number of occurrences. The three strongest NP properties and experimental condition interactions in the networks are highlighted in red.

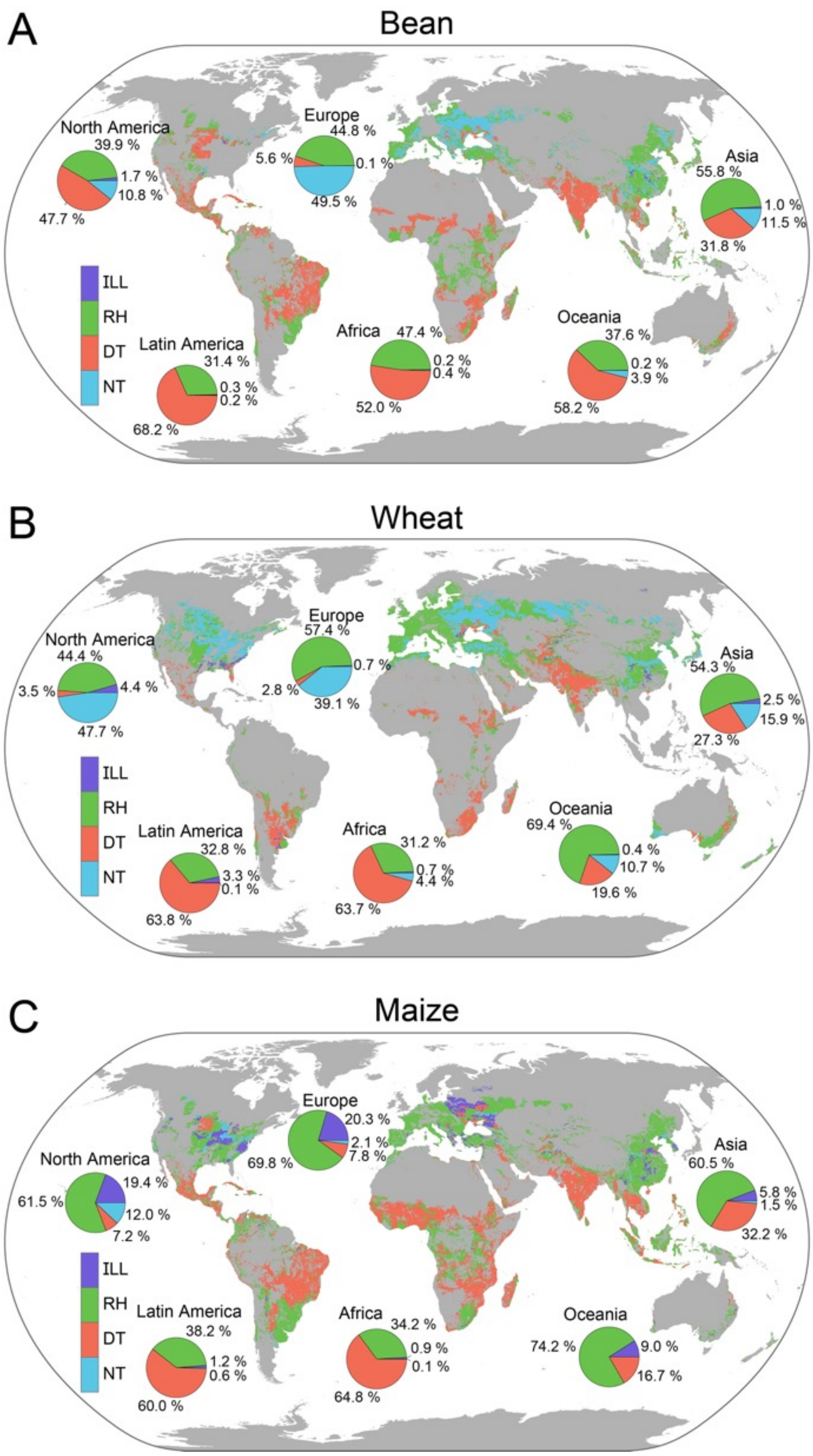

Fig. S25. Contribution of important environmental factors regulating crop root length influenced by Fe_2O_3 NP (50 mg) during the seedling stage. (A) Bean; (B) wheat and (C) maize. Environmental factors include the illumination intensity (ILL), relative humidity (RH), daytime temperature (DT) and night temperature (NT). The map has a spatial resolution of 5 arcmin, which is approximately 10 km × 10 km at the equator.

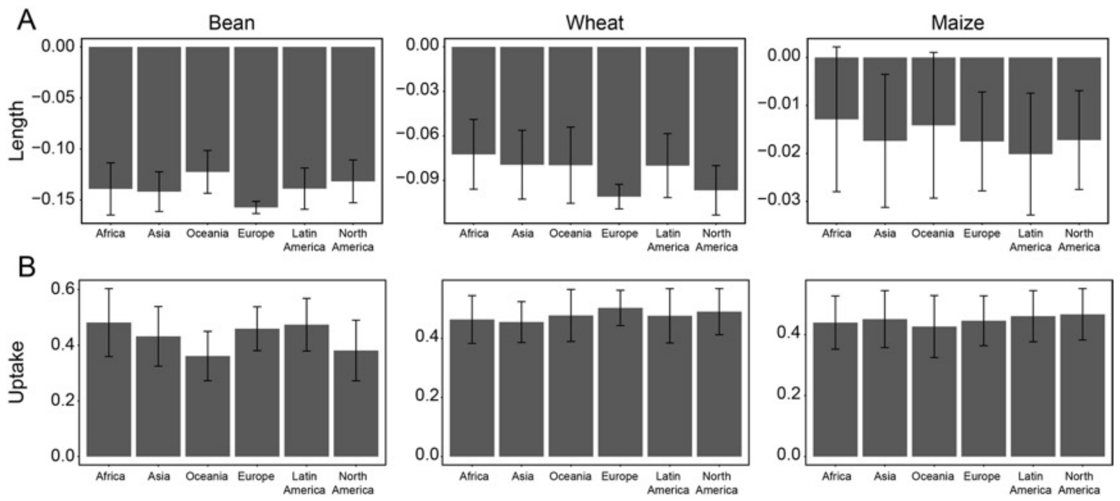

Fig. S26. Prediction of plant root length and NP uptake at continental scales with laboratory and field data. (A) Continental average of plant root length values; (B) continental average of plant root uptake values. The values were transformed and are unitless and are shown in the *Methods*.

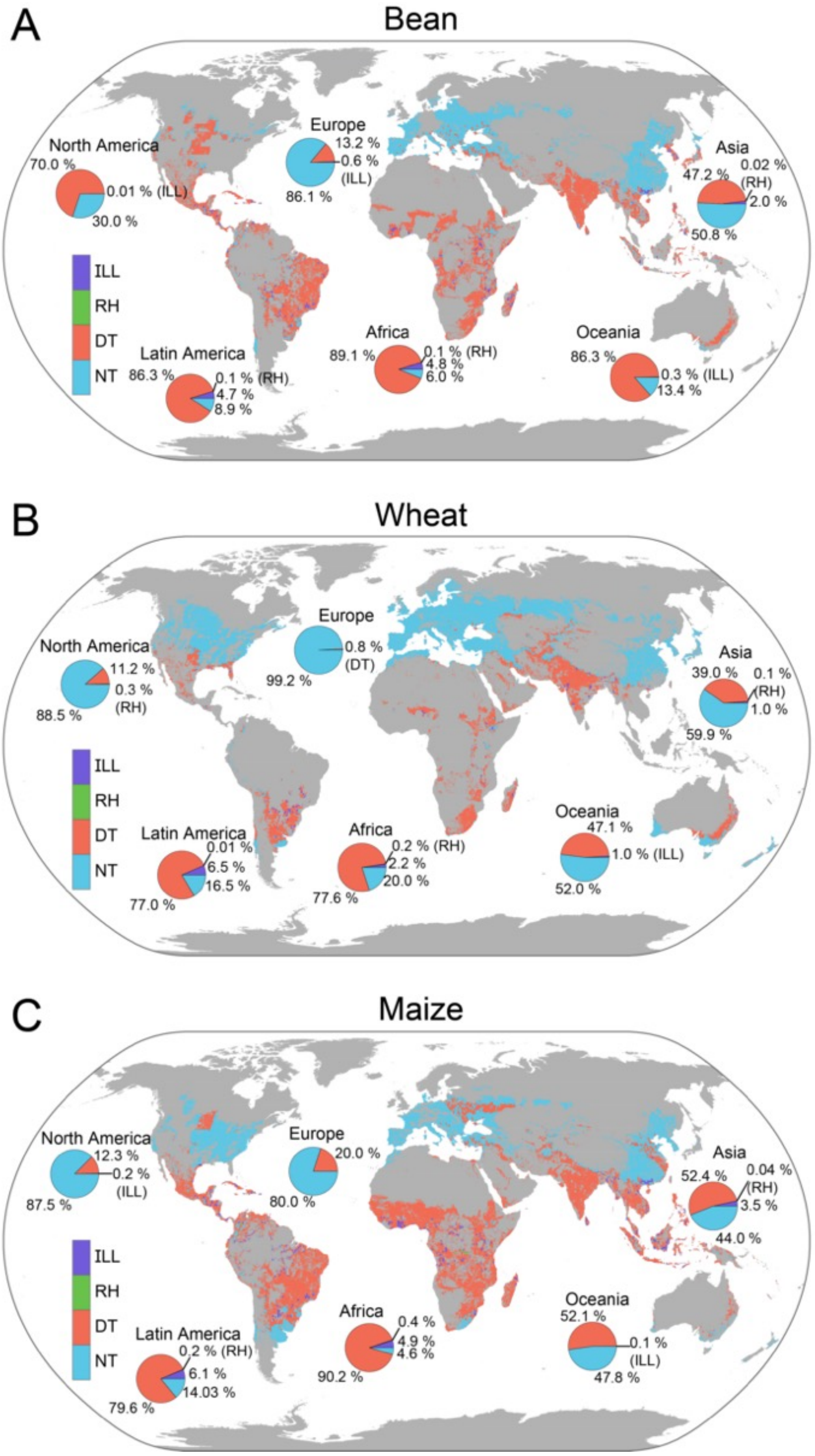

Fig. S27. Contribution of important environmental factors regulating crop root uptake influenced by ZnO NPs (50 mg) during the seedling stage. (A) Bean; (B) wheat and (C) maize. Environmental factors include the illumination intensity (ILL), relative humidity (RH), daytime temperature (DT) and night temperature (NT). The map has a spatial resolution of 5 arcmin, which is approximately 10 km × 10 km at the equator.

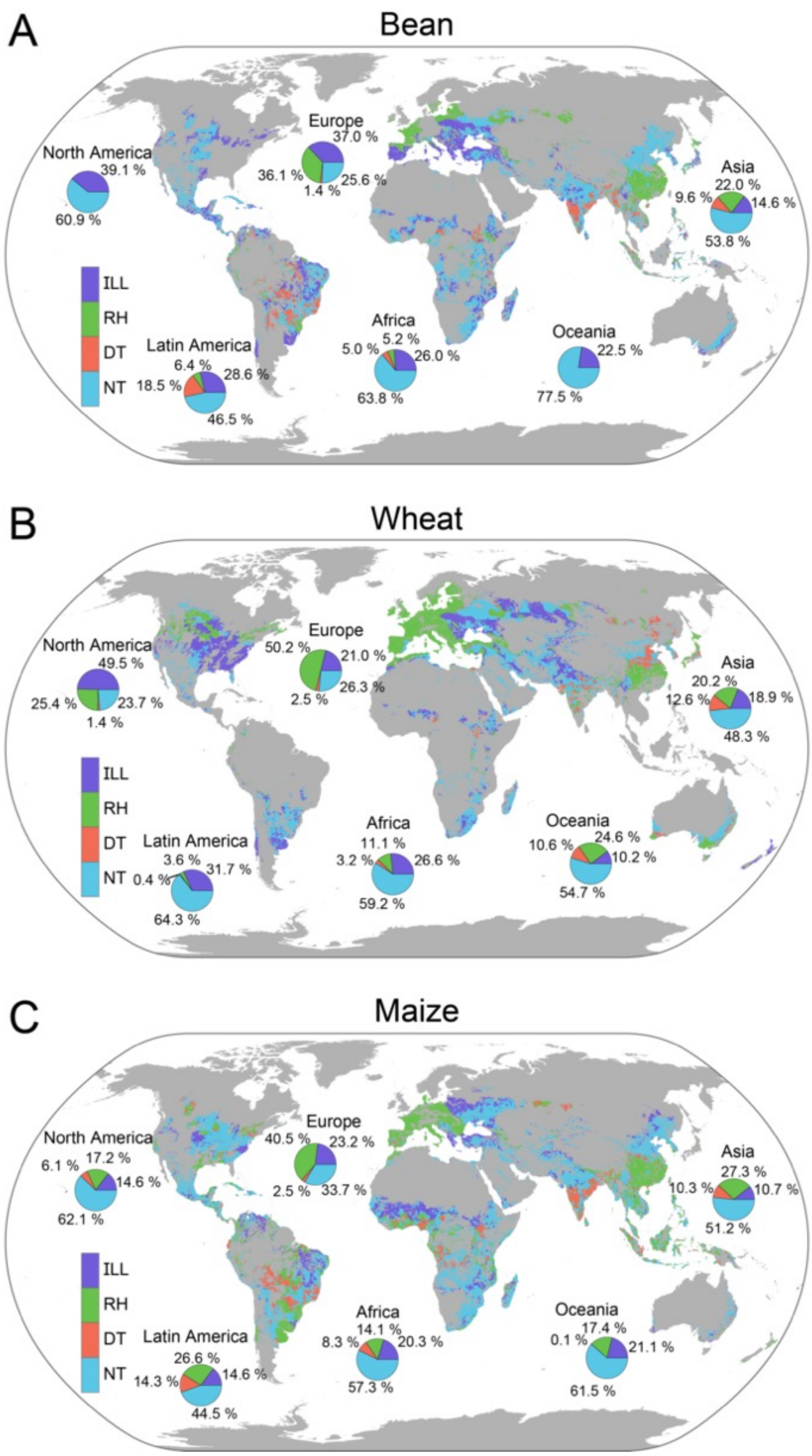

Fig. S28. Contribution of important environmental factors regulating oxidative stress of the crop leaves induced by ZnO NPs (50 mg) during the seedling stage. (A) Bean; (B) wheat and (C) maize. Environmental factors include the illumination intensity (ILL), relative humidity (RH), daytime temperature (DT) and night temperature (NT). The map has a spatial resolution of 5 arcmin, which is approximately 10 km × 10 km at the equator.

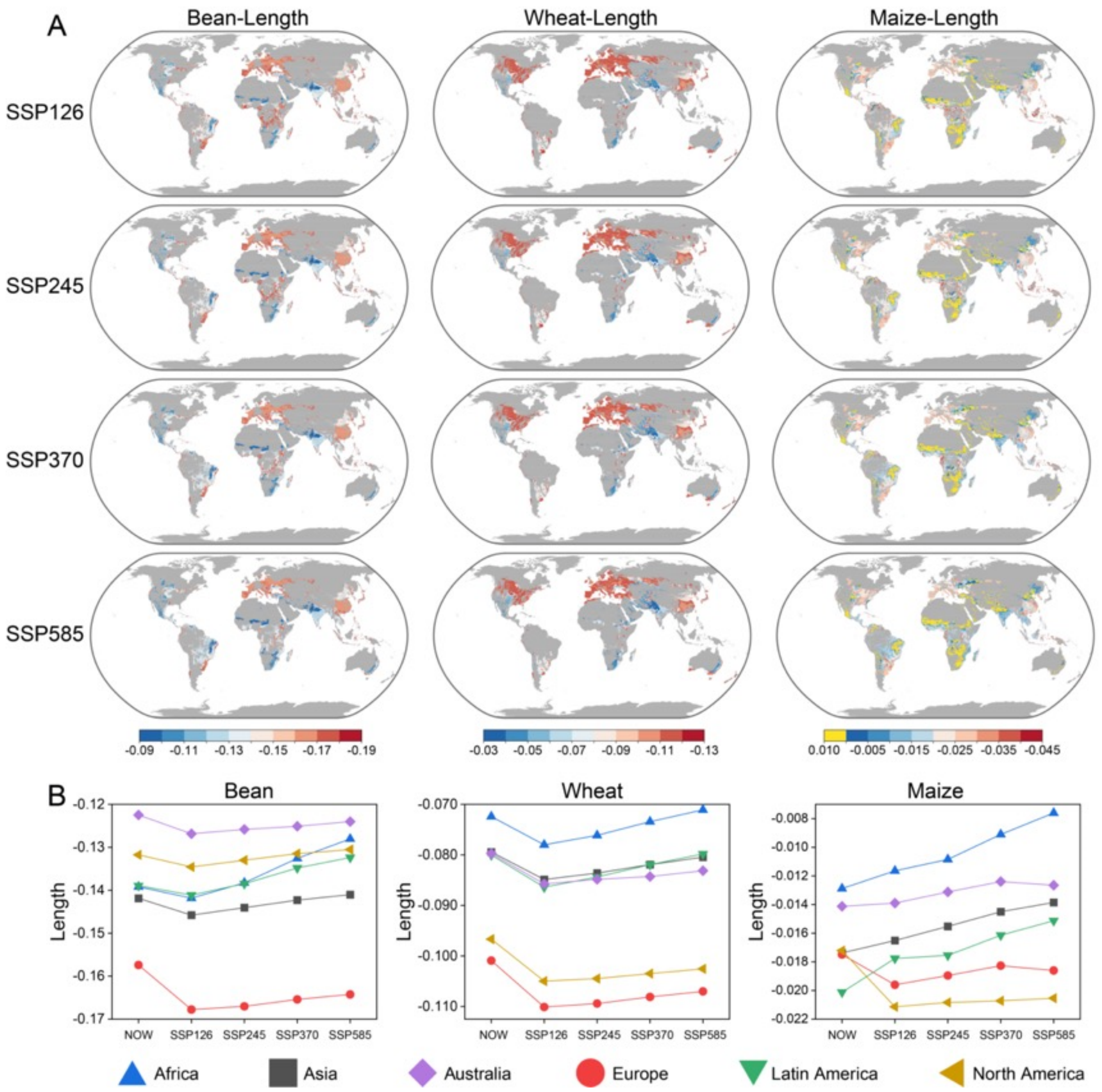

Fig. S29. Spatial patterns of plant root length projected under four future scenarios in 2080-2100 using models with laboratory and field data. (A) The effect of 50 mg Fe_2O_3 NPs on the length of the crop roots during the planting period. The four future scenarios are SSP126, SSP245, SSP370, and SSP585, from top to bottom. The map has a spatial resolution of 5 arcmin, corresponding to approximately 10 km × 10 km at the equator. (B) Continental average of the plant root lengths under the four SSP scenarios and now.

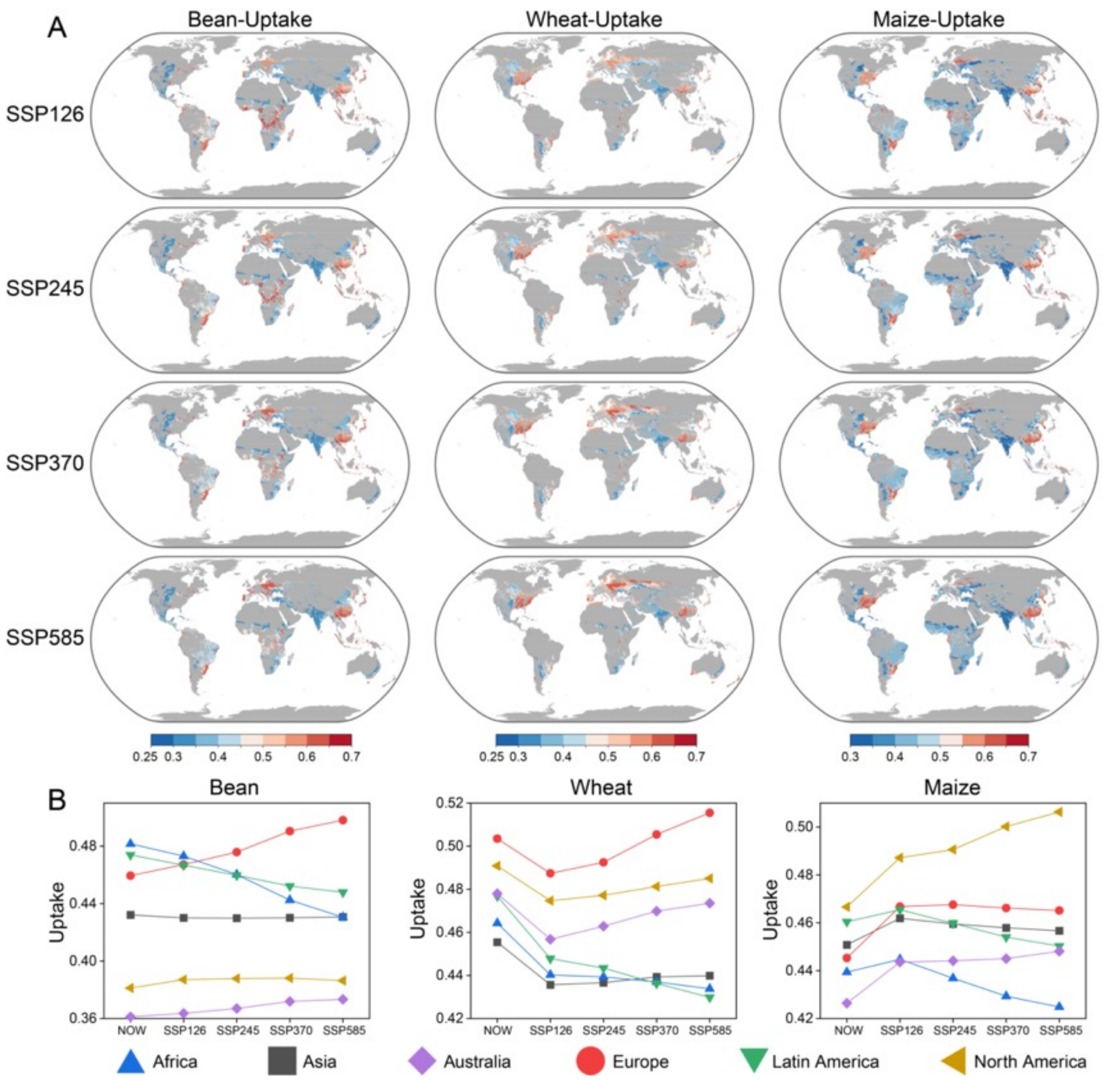

Fig. S30. Spatial patterns of plant root uptake of NPs projected under four future scenarios in 2080-2100 using models with laboratory and field data. (A) NP uptake by crop roots under 50 mg ZnO NPs during the planting period. The four future scenarios are SSP126, SSP245, SSP370, and SSP585, from top to bottom. The map has a spatial resolution of 5 arcmin, which is approximately 10 km × 10 km at the equator. (B) Continental average of plant root uptake values of NPs under the four SSP scenarios and now.

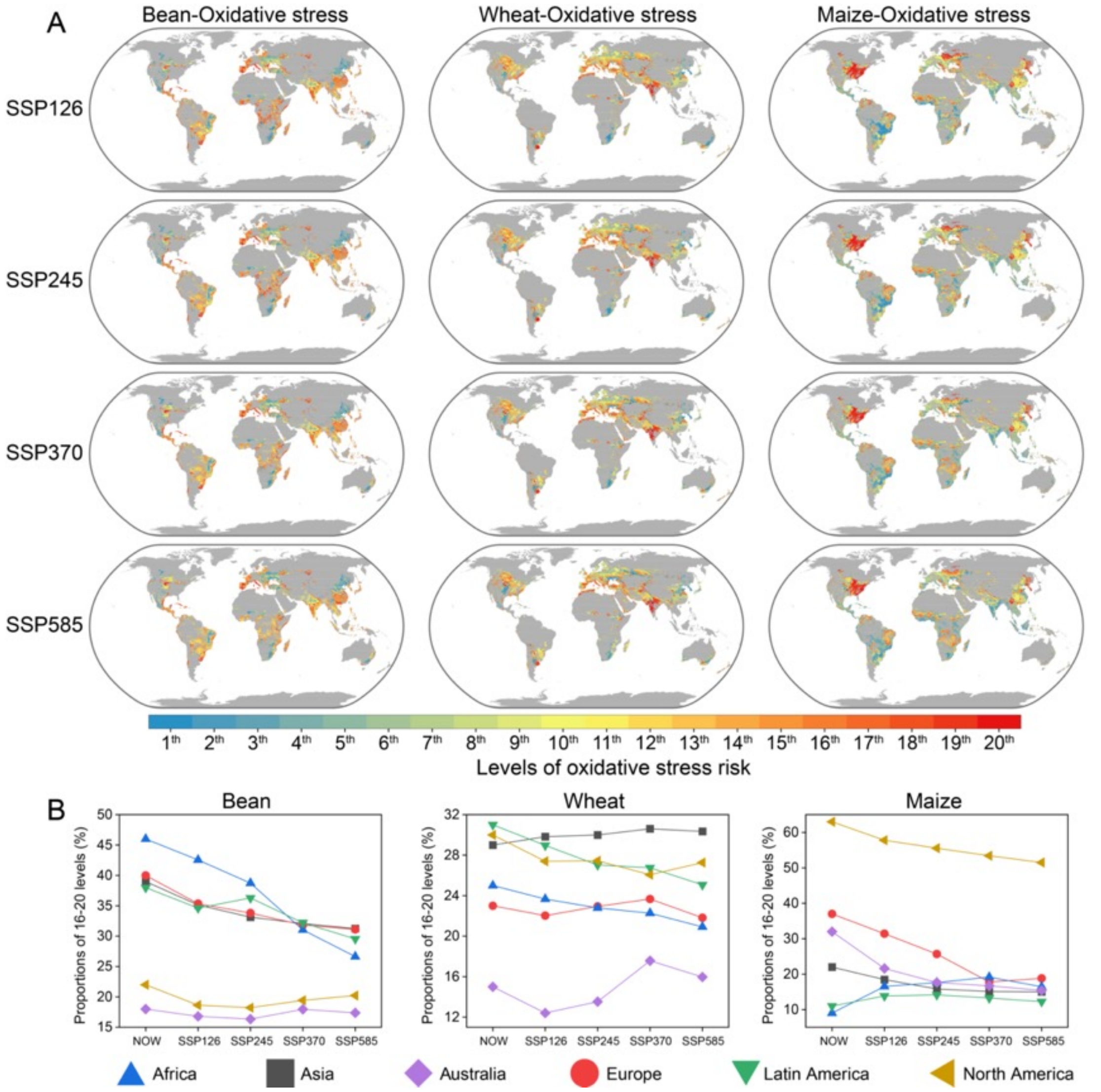

Fig. S31. Spatial patterns of plant oxidative stress risk levels caused by NPs projected under four future scenarios in 2080-2100 using models with laboratory and field data. (A) The effect of 50 mg ZnO NPs on the oxidative stress of the leaves of crops during the planting period. The map has a spatial resolution of 5 arcmin, which is approximately 10 km × 10 km at the equator. (B) Proportions of plant leaf oxidative stress at the 16th-20th levels in the related areas under the four SSP scenarios and now.

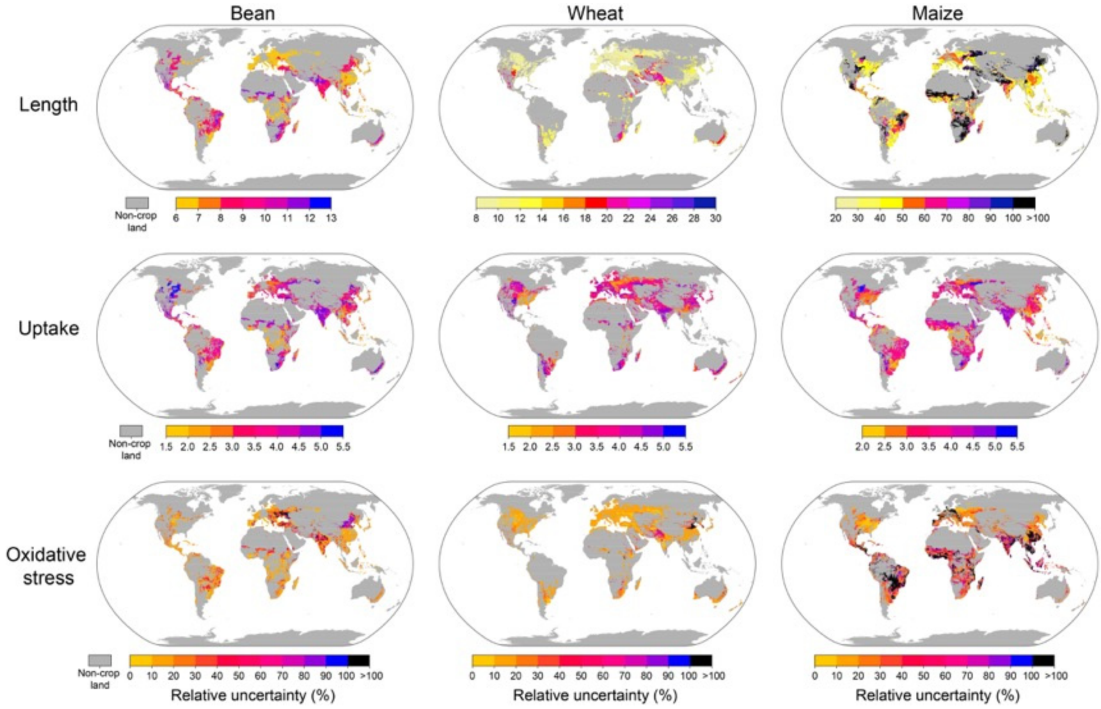

Fig. S32. Uncertainty analysis of projected global plant length, uptake and oxidative stress prediction using models with laboratory and field data.

Supplementary Tables

Table S1. Dataset supplementary description of NP types and shapes.

Features	Data distribution	Features	Data distribution
**NP types**	n=16	**NP shapes**	n=6
ZnO	31.43%	Sphere	44.97%
Ag	9.88%	Rod	35.95%
CuO	9.88%	Irregular	8.69%
Graphene	7.75%	Dot	4.77%
Fe_2O_3	7.24%	Sheet	2.98%
TiO_2	6.30%	Tube	2.64%
CeO_2	6.05%		
Fe_3O_4	5.37%		
PS	5.20%		
Al_2O_3	2.73%		
MWCNTs	2.64%		
nZVI	2.30%		
Cu	1.36%		
MgO	0.77%		
SiO_2	0.77%		
Fe	0.34%		

Table S2. Performance of the machine learning models.

		R^2					RMSE				
Subset	Spilt	RF	XGB	ANN	SVM	RF-SBS	RF	XGB	ANN	SVM	RF-SBS
Length	Training	0.984±0.001	0.999±0.001	0.870±0.009	0.821±0.010	0.984±0.001	0.051±0.001	0.004±0.000	0.125±0.004	0.145±0.002	0.051±0.001
	Test	0.849±0.081	0.839±0.112	0.776±0.069	0.741±0.130	0.854±0.072	0.134±0.029	0.135±0.042	0.167±0.026	0.171±0.015	0.133±0.026
DW	Training	0.987±0.002	0.999±0.001	0.838±0.011	0.808±0.018	0.987±0.002	0.054±0.004	0.005±0.001	0.169±0.005	0.181±0.007	0.056±0.003
	Test	0.832±0.152	0.842±0.136	0.754±0.105	0.711±0.126	0.823±0.151	0.166±0.054	0.159±0.060	0.205±0.030	0.233±0.047	0.172±0.051
RS	Training	0.986±0.003	0.999±0.001	0.926±0.014	0.914±0.011	0.987±0.002	0.045±0.003	0.002±0.000	0.095±0.007	0.124±0.007	0.045±0.003
	Test	0.852±0.177	0.864±0.144	0.806±0.102	0.828±0.173	0.852±0.180	0.104±0.047	0.114±0.057	0.109±0.062	0.114±0.041	0.100±0.048
APX	Training	0.974±0.002	0.999±0.001	0.806±0.013	0.734±0.011	0.974±0.002	0.086±0.003	0.004±0.001	0.192±0.005	0.225±0.006	0.087±0.003
	Test	0.750±0.121	0.716±0.106	0.639±0.138	0.626±0.088	0.739±0.123	0.218±0.045	0.240±0.044	0.286±0.034	0.271±0.053	0.222±0.049
H_2O_2	Training	0.986±0.001	0.999±0.001	0.859±0.015	0.764±0.013	0.985±0.001	0.058±0.001	0.003±0.000	0.158±0.007	0.199±0.005	0.060±0.002
	Test	0.842±0.115	0.847±0.086	0.662±0.117	0.652±0.150	0.834±0.127	0.153±0.046	0.158±0.040	0.237±0.031	0.226±0.039	0.155±0.048
MDA	Training	0.981±0.001	0.999±0.001	0.848±0.020	0.781±0.012	0.980±0.001	0.054±0.002	0.004±0.000	0.130±0.007	0.150±0.004	0.055±0.002
	Test	0.763±0.104	0.746±0.127	0.626±0.171	0.665±0.153	0.754±0.113	0.154±0.029	0.161±0.032	0.192±0.038	0.183±0.038	0.156±0.030
SOD	Training	0.980±0.001	0.999±0.001	0.853±0.027	0.760±0.025	0.980±0.002	0.043±0.001	0.004±0.001	0.099±0.007	0.122±0.004	0.043±0.001
	Test	0.807±0.105	0.773±0.084	0.644±0.139	0.568±0.181	0.799±0.104	0.123±0.038	0.134±0.030	0.153±0.031	0.172±0.040	0.125±0.038
Chl *a*	Training	0.982±0.001	0.999±0.001	0.879±0.009	0.838±0.011	0.982±0.001	0.053±0.002	0.004±0.001	0.116±0.006	0.132±0.003	0.053±0.002
	Test	0.832±0.092	0.830±0.101	0.747±0.080	0.691±0.131	0.820±0.094	0.139±0.037	0.140±0.039	0.161±0.032	0.180±0.027	0.144±0.038
Chl *b*	Training	0.978±0.002	0.999±0.001	0.868±0.015	0.789±0.008	0.978±0.002	0.063±0.003	0.004±0.001	0.130±0.006	0.160±0.003	0.063±0.002
	Test	0.793±0.091	0.771±0.152	0.614±0.135	0.651±0.109	0.788±0.085	0.161±0.037	0.166±0.057	0.231±0.047	0.204±0.028	0.162±0.035
Uptake	Training	0.988±0.001	0.999±0.001	0.826±0.009	0.738±0.007	0.988±0.001	0.065±0.002	0.006±0.001	0.214±0.005	0.256±0.002	0.066±0.002
	Test	0.884±0.048	0.884±0.060	0.662±0.064	0.563±0.088	0.886±0.049	0.176±0.027	0.172±0.038	0.288±0.029	0.323±0.027	0.177±0.027
TF	Training	0.985±0.001	0.999±0.001	0.897±0.013	0.856±0.013	0.985±0.001	0.126±0.005	0.004±0.001	0.280±0.017	0.337±0.025	0.127±0.004
	Test	0.847±0.076	0.796±0.133	0.718±0.183	0.840±0.091	0.846±0.074	0.339±0.132	0.400±0.128	0.457±0.174	0.366±0.201	0.339±0.133
RCF	Training	0.980±0.004	1.000±0.000	0.926±0.003	0.743±0.027	0.982±0.003	0.774±0.035	0.002±0.000	1.268±0.249	2.833±0.208	0.775±0.036
	Test	0.828±0.114	0.795±0.189	0.877±0.088	0.641±0.234	0.821±0.106	2.264±1.018	2.037±0.970	1.965±0.862	3.006±1.442	2.269±1.050
SCF	Training	0.970±0.006	1.000±0.000	0.916±0.025	0.664±0.035	0.972±0.006	0.713±0.037	0.003±0.001	1.032±0.157	2.182±0.193	0.704±0.032
	Test	0.831±0.158	0.697±0.341	0.735±0.187	0.736±0.203	0.865±0.104	1.809±1.228	2.0248±1.828	1.768±0.780	2.199±1.781	1.726±1.105

Table S3. List of environmental data for bean growth at the continental scale.

Region	Acidic soil (%)	Neutral soil (%)	Alkaline soil (%)	Daytime Temperature (°C)	Night Temperature (°C)	Illumination Intensity (μmol·$m^{-2}s^{-1}$)	Relative Humidity (%)
Africa	69.35	23.95	6.70	31.40±6.59	18.89±4.30	290.06±44.75	67.15±20.07
Asia	43.13	33.24	23.63	23.31±11.79	10.80±12.40	234.83±56.78	64.32±15.52
Australia	29.96	45.70	24.34	32.95±5.26	12.63±3.70	312.45±17.48	57.17±9.61
Europe	45.06	48.14	6.80	17.41±7.82	4.69±5.96	230.95±29.39	66.53±5.97
Latin America	83.45	12.71	3.83	31.83±5.79	19.07±4.78	278.67±43.11	71.33±12.68
North America	28.65	59.25	12.10	29.39±5.38	9.95±3.34	315.48±22.86	57.35±11.12

The field global gridded dataset of daytime and night temperatures (data source, https://www.neo.gsfc.nasa.gov/), soil types (data source, https://www.soilgrids.org/), illumination density (data source, https://www.neo.gsfc.nasa.gov/) and relative humidity (data source, https://www cds.climate.copernicus.eu/) was used.

Table S4. List of environmental data for wheat growth at the continental scale.

**Region**	**Acidic soil (%)**	**Neutral soil (%)**	**Alkaline soil (%)**	**Daytime Temperature (°C)**	**Night Temperature (°C)**	**Illumination Intensity (μmol·m^{-2}s^{-1})**	**Relative Humidity (%)**
Africa	49.26	31.92	18.82	31.84±8.00	16.02±5.72	302.67±56.55	62.81±16.26
Asia	21.71	39.31	38.98	21.16±15.00	6.16±13.04	233.11±72.99	57.16±17.47
Australia	47.33	35.94	16.74	25.29±7.49	10.82±4.46	221.80±52.99	62.13±12.76
Europe	56.83	38.67	4.51	13.25±7.77	1.22±5.23	196.89±48.93	68.11±8.93
Latin America	55.41	36.47	8.12	30.08±5.32	14.90±4.74	323.34±37.09	67.76±12.35
North America	45.16	46.33	8.51	15.84±9.28	0.21±7.04	250.55±42.99	61.26±9.46

The field global gridded dataset of daytime and night temperatures (data source, https://www.neo.gsfc.nasa.gov/), soil types (data source, https://www.soilgrids.org/), illumination density (data source, https://www.neo.gsfc.nasa.gov/) and relative humidity (data source, https://www cds.climate.copernicus.eu/) was used.

Table S5. List of environmental data for maize growth at the continental scale.

**Region**	**Acidic soil (%)**	**Neutral soil (%)**	**Alkaline soil (%)**	**Daytime Temperature (°C)**	**Night Temperature (°C)**	**Illumination Intensity ($\mu mol \cdot m^{-2} s^{-1}$)**	**Relative Humidity (%)**
Africa	73.50	21.07	5.43	33.91±6.26	19.35±4.54	294.70±43.68	59.09±18.10
Asia	45.45	29.01	25.55	25.94±9.59	13.00±10.11	250.45±58.16	63.31±17.05
Australia	46.43	37.81	15.75	26.79±7.18	10.18±4.03	289.20±33.58	60.05±12.96
Europe	44.34	49.33	6.32	18.15±9.40	4.36±6.24	224.48±76.73	65.51±10.59
Latin America	77.52	17.87	4.61	30.55±6.07	17.59±5.85	272.13±47.22	68.62±13.68
North America	55.55	38.29	6.16	18.95±7.46	2.59±5.80	246.52±39.03	61.36±7.96

The field global gridded dataset of daytime and night temperatures (data source, https://www.neo.gsfc.nasa.gov/), soil types (data source, https://www.soilgrids.org/), illumination density (data source, https://www.neo.gsfc.nasa.gov/) and relative humidity (data source, https://www cds.climate.copernicus.eu/) was used.

Table S6. Parameters of plant responses and uptake/transport indicators of NPs to represent the development potential of nanoengineered agriculture.

		Parameters	Unit
Plant responses	Growth	Length	cm
		Dry weight	g
		Root-shoot ratio	unitless
	Oxidative stress	Ascorbate peroxidase	%*
		Hydrogen peroxide	%*
		Malondialdehyde	%*
		Superoxide dismutase	%*
	Chlorophyll	Chlorophyll *a*	%*
		Chlorophyll *b*	%*
NPs uptake/transport	Uptake	Uptake	mg/g
	Transport	TF	unitless
		RCF	unitless
		SCF	unitless

*, "%" means that the value of extracted data was processed according to the control group, and the control group was set to 100%.

Table S7. List of abbreviations and the corresponding full terms.

Full name	Abbreviation
Nanoparticles	NPs
Macromolecular compound	MC
Component 1	Com.1
Component 2	Com.2
Granular	Dim0
One-dimensional	Dim1
Two-dimensional	Dim2
Exposed pathway	EP
Measured tissue	MT
Size measured by transmission electron microscopy	$size_{TEM}$
Size measured by dynamic light scattering	$size_{DLS}$
Growing stage	GS
Daytime temperature	DT
Night temperature	NT
Dry weight	DW
Root/shoot ratio	RS
Ascorbate peroxidase	APX
Hydrogen per oxide	H_2O_2
Malondialdehyde	MDA
Superoxide dismutase	SOD
Chlorophyll *a*	Chl *a*
Chlorophyll *b*	Chl *b*
Transport factor	TF
Root concentration factor	RCF
Shoot concentration factor	SCF
Root mean square error	RMSE
Mean square error increase	MSE increase

Table S8. List of global prediction parameter settings.

Feature	Length	Uptake	APX	H_2O_2	MDA	SOD
NP type	Fe_2O_3	ZuO	ZnO	ZnO	ZnO	ZnO
$Size_{TEM}$	20	68	68	68	68	68
$Size_{DLS}$	80	278	278	278	278	278
Purity	99	99	99	99	99	99
Shape	Sphere	Sphere	Sphere	Sphere	Sphere	Sphere
Zeta Potential	-13.2	-13.7	-13.7	-13.7	-13.7	-13.7
Species	/	/	/	/	/	/
Exposed pathway	Seed	Seed	Seed	Seed	Seed	Seed
Measured tissue	Root	Root	Leaf	Leaf	Leaf	Leaf
Age of plants at treatment	0	0	0	0	0	0
Growing stages	Germination	Germination	Germination	Germination	Germination	Germination
Cultured	Soil	\	\	\	\	\
Category	\	\	\	\	\	\
Total dose	50	50	50	50	50	50
Duration	21	21	21	21	21	21
Photoperiod	16	16	16	16	16	16
Illumination density	\	\	\	\	\	\
Humidity	\	\	\	\	\	\
Daytime temperature	\	\	\	\	\	\
Night temperature	\	\	\	\	\	\
